# Identification and verification of methylenetetrahydrofolate dehydrogenase 1-like protein as the binding target of natural product pseudolaric acid A

**DOI:** 10.1007/s13659-025-00502-1

**Published:** 2025-04-02

**Authors:** Haoqi Dong, Xinni Yang, Peiying Wang, Weiya Huang, Liang Zhang, Song Song, Jiangxin Liu

**Affiliations:** 1 State Key Laboratory of Phytochemistry and Natural Medicines, Kunming Institute of Botany, Chinese Academy of Sciences, Kunming, 650201 China; 2 University of Chinese Academy of Sciences, Beijing, 100049 China; 3 Yunnan University, Kunming, 650500 China; 4 State Key Laboratory of Natural and Biomimetic Drugs, School of Pharmaceutical Sciences, Peking University, Beijing, 100191 China

**Keywords:** Natural product, Target identification, Chemical proteomics, Target verification

## Abstract

Natural product pseudolaric acid A (PAA), the main bioactive component from Traditional Chinese Medicine *Pseudolarix cortex* (“tujingpi”), is a promising anticancer agent. However, its potential molecular targets are not clear and this hinders its development. In this study, chemical proteomics approaches including activity-based protein profiling (ABPP) and drug affinity responsive target stability (DARTS) technology, followed by quantitative proteomics, were combined to reveal the target of PAA. Target validation was performed by NMR techniques and surface plasmon resonance. Methylenetetrahydrofolate dehydrogenase 1-like (MTHFD1L) was identified and further confirmed to be the target of PAA. The direct interaction and binding mode between MTHFD1L and PAA were elaborated. PAA induced the accumulation of the reactive oxygen species (ROS) which mediates the antitumor effect. Transcriptome and network pharmacology analysis reveals the effects of PAA on the gene expressions of the associated pathways. Taken together, our findings proposed a new target that could be used for structure-based rational design and modifications of PAA.

**Graphical abstract:**

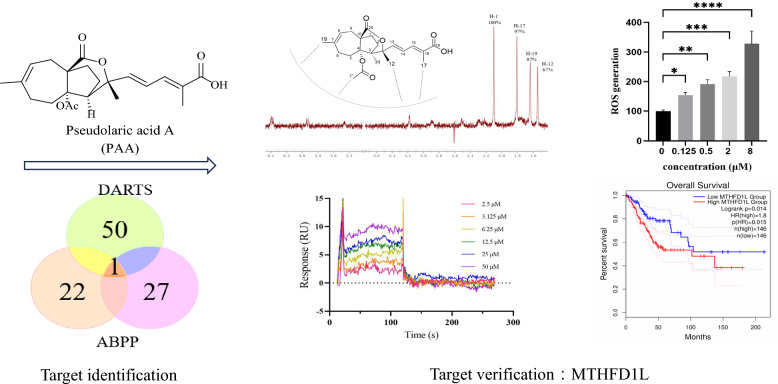

**Supplementary Information:**

The online version contains supplementary material available at 10.1007/s13659-025-00502-1.

## Highlights

Different approaches including ABPP and DARTS were combined to reveal the target of natural product PAA.

MTHFD1L is the new protein target of PAA.

The direct interaction between PAA and MTHFD1L is demonstrated.

MTHFD1L exhibits significant association with unfavorable prognostic outcome in CESC cancer.

**Supplementary Information:**

The online version contains supplementary material available at 10.1007/s13659-025-00502-1.

## Introduction

Natural products (NP) have been an important source of drug discovery and development to target various diseases [1]. Despite the remarkable achievements of natural products, the major bottleneck of drug development from natural product mainly remains to the difficulties in comprehensively understanding the mechanism of action (MOA) of NP [2, 3]. NP exert their pharmacological functions through binding to biological macro-molecules, interacting with different protein network. Thus, understanding the protein targets is the initial critical step and the foundation of NP-based natural medicine development [4, 5]. It could also lead to the identification of the off-target side effects and potential toxicities of natural medicine [6]. Thus, target identification and interaction investigation between the NP and targets are essential for the potential development of natural products [7].

Pseudolaric acid A (PAA), a diterpenoid isolated from traditional Chinese materia medica *Pseudolarix cortex* (“tujingpi”), has been used to treat fungal skin infections since the seventeenth century. As the main bioactive constituent of *Pseudolarix cortex*, it displays primary pharmacological activities, including antifungal, anti-fertility, and anti-cancer activity [8, 9]. Although various biological activities have been reported for quite some time, the precise molecular targets of PAA and mechanisms underlying the multiple bioactivities remain elusive. Previously, we discovered that PAA exhibited HSP90 inhibitory activity [10]. However, the inhibition activity against HSP90 shows significant deviation with the activities against most cancer cells (0.60–6.16 μM), indicating that other protein partners involve and play important roles in the anticancer activity of PAA. Thus, identifying other protein targets is an important issue and need to be solved.

Chemical proteomics approach including activity-based protein profiling (ABPP), association with quantitative proteomics has been widely utilized in target identification of bioactive natural products [11]. However, the modified molecular probe for ABPP approach, with the addition of an exogenous group, may interfere with the pharmacological activity of natural products and potential binding proteins, introducing bias to the results [12]. Therefore, non-labeling chemical proteomics approach, for example, drug affinity responsive target stability (DARTS) strategy is also applied to investigate the target proteins of NPs [13, 14]. DATRS uses native and unmodified small molecules, which is relatively simple [15]. Each strategy has its own advantages and drawbacks. Therefore, the strategy of integrating ABPP and DARTS methods together broadly screens the potential drug targets and could increase the confidence of results and decrease the risk of false positive.

In this study, we collectively applied different approaches to identify methylenetetrahydrofolate dehydrogenase 1 like (MTHFD1L) as one of the potential target proteins of PAA. MTHFD1L was further biochemically validated and the direct interaction between MTHFD1L and PAA was investigated. Transcriptome analysis and bioinformatic analysis support the deeper understanding of gene MTHFD1L functions in cervical & endocervical cancer (CESC). Taken together, this study discovered and highlighted the direct interaction between MTHFD1L and PAA. The newly identified target could be used to guide the structure-based rational design and modifications of PAA.

## Results and discussion

### Potential binding proteins of PAA identified by DARTS method

**Fig. 1:**
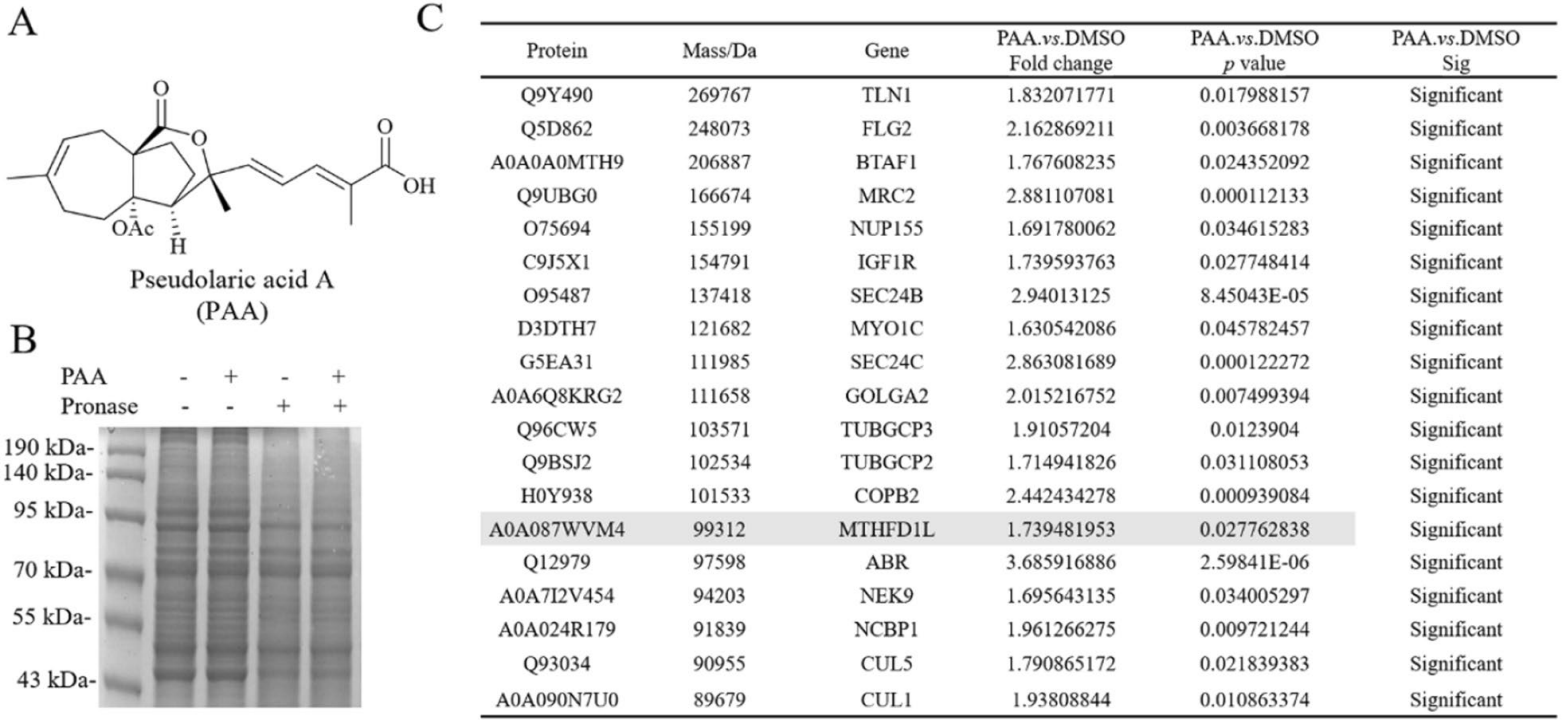
Potential binding proteins of PAA identified by DARTS. **A** Chemical structure of PAA. **B** Coomassie stained gel showing proteins with/without treatment of pronase. The HeLa cell lysate was treated with PAA and different concentrations of pronase. Different ratios of PAA to pronase were optimized, and 1:100 (pronase: protein sample) was chosen for the following procedures. **C** Part of the putative PAA interacting proteins, which has been identified with the following two criteria: fold change of different groups (Fold Change > 1.5) and *p* value < 0.05

Considering that PAA exhibited significant antitumor activity in HeLa cell (cervical cancer cell line) (IC_50_ = 2.92 ± 0.36 µM), we conducted the label free target identification method based on shift in the thermal stability of protein, which is DARTS-centered technology to explore the protein targets of natural product PAA in HeLa cells. Different ratios of pronase to proteins were applied to optimize the hydrolysis conditions (Fig. 1B). When small compounds bind with proteins, this interaction stabilizes the target proteins, thus making them resistant to protease. The LC–MS/MS analysis of the digested peptide samples and Mascot database search could identify the potential binding proteins. The list of putative PAA interacting partners was refined by only including proteins protected by PAA. Results from two repeats of the DARTS experiments showed that there were 42 overlapping proteins, both of which were up-regulated in the PAA and pronase-treated group, as the result of PAA induced protein stabilization and reduction in proteolysis susceptibility. Part of the potential binding proteins was shown in Fig. 1C. The initial identification of protein targets using DARTS chemical proteomics approach exhibited 42 potential binding partners of PAA (Table S1).

### Potential binding proteins of PAA identified by ABPP method

**Fig. 2:**
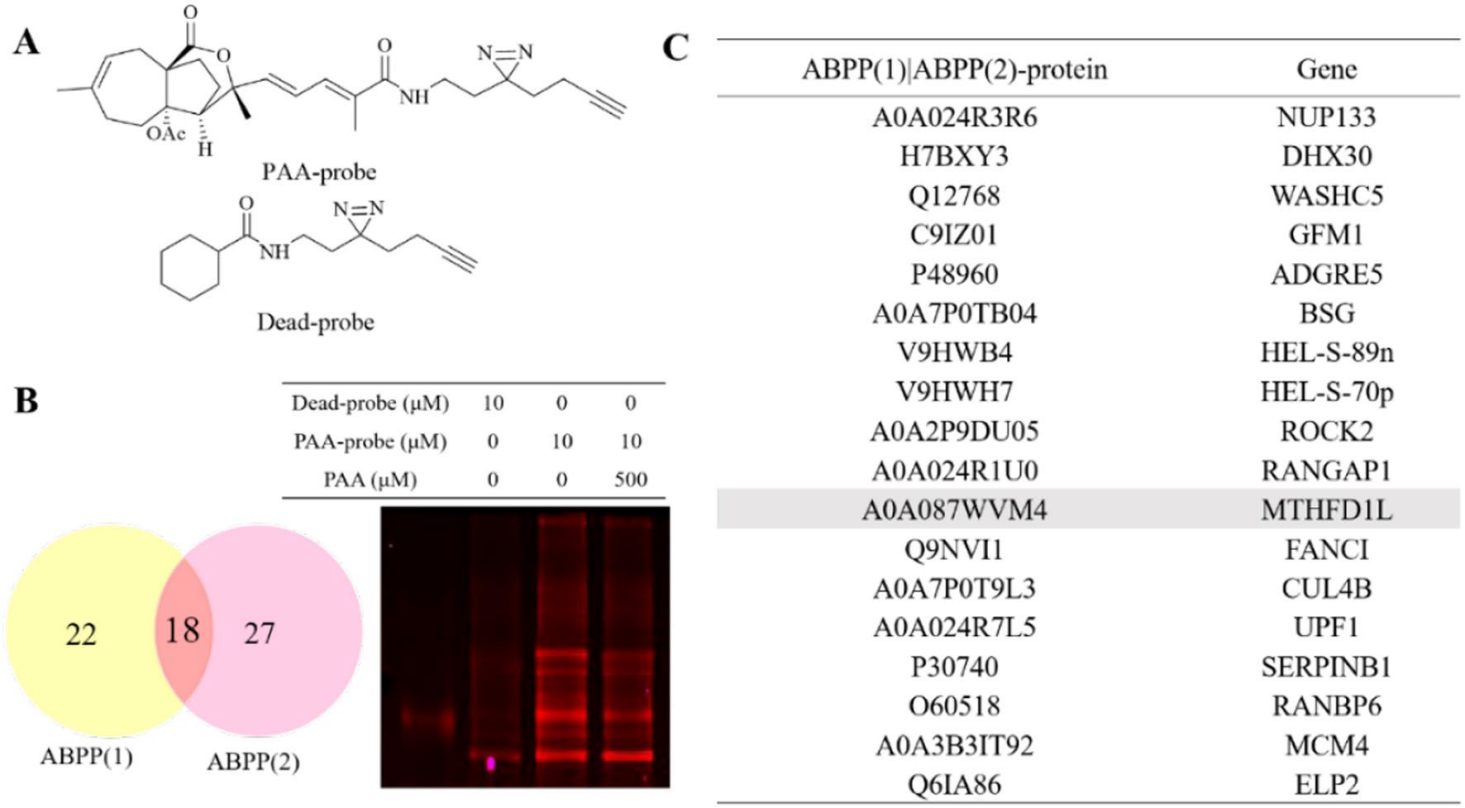
Potential binding proteins of PAA identified by ABPP. **A** Chemical structures of PAA-probe and dead-probe (control probe). **B** Vanne map shows the overlapping proteins through two repeats of ABPP assay (left) and competition assay of the PAA-probe and PAA alone, visualized by in-gel fluorescence scanning at 533 nm (right). **C** Collected protein and gene information of the overlapped binding partners of PAA from two repeats of the ABPP assay

To further refine the potential binding proteins of PAA, we further utilized the activity-based protein mass spectrometry (ABPP) strategy using the PAA-probe designed in previous study [10]. PAA probe exhibited similar biological activity as PAA (Fig. 2A). Dead probe was set as a negative control. The HeLa cell proteomes were divided into three groups to explore PAA interacting protein, including control group (10 μM dead probe), PAA-probe group (10 μM PAA-probe), Competition group (500 μM PAA first and followed by 10 μM PAA probe). When bands from SDS-PAGE displayed no fluorescent intensity in the control group, the highest fluorescent intensity in the probe-treated group, and weaker fluorescent intensity in the competition group, they are considered to exhibit apparent competitive effects. Bands ranging from 40–55 kDa, 70–80 kDa, and 90–130 kDa were cut and proceeded with quantification sequencing (Fig. 2B). The ABPP workflow was repeated twice to reproduce the results. 18 potential binding proteins were overlapped in the two repeated assays. The detailed information of the overlapped proteins was shown in Fig. 2C (Table S2).

### Most potential binding target of PAA and its association with cervical & endocervical cancer through bioinformatic analysis

**Fig. 3:**
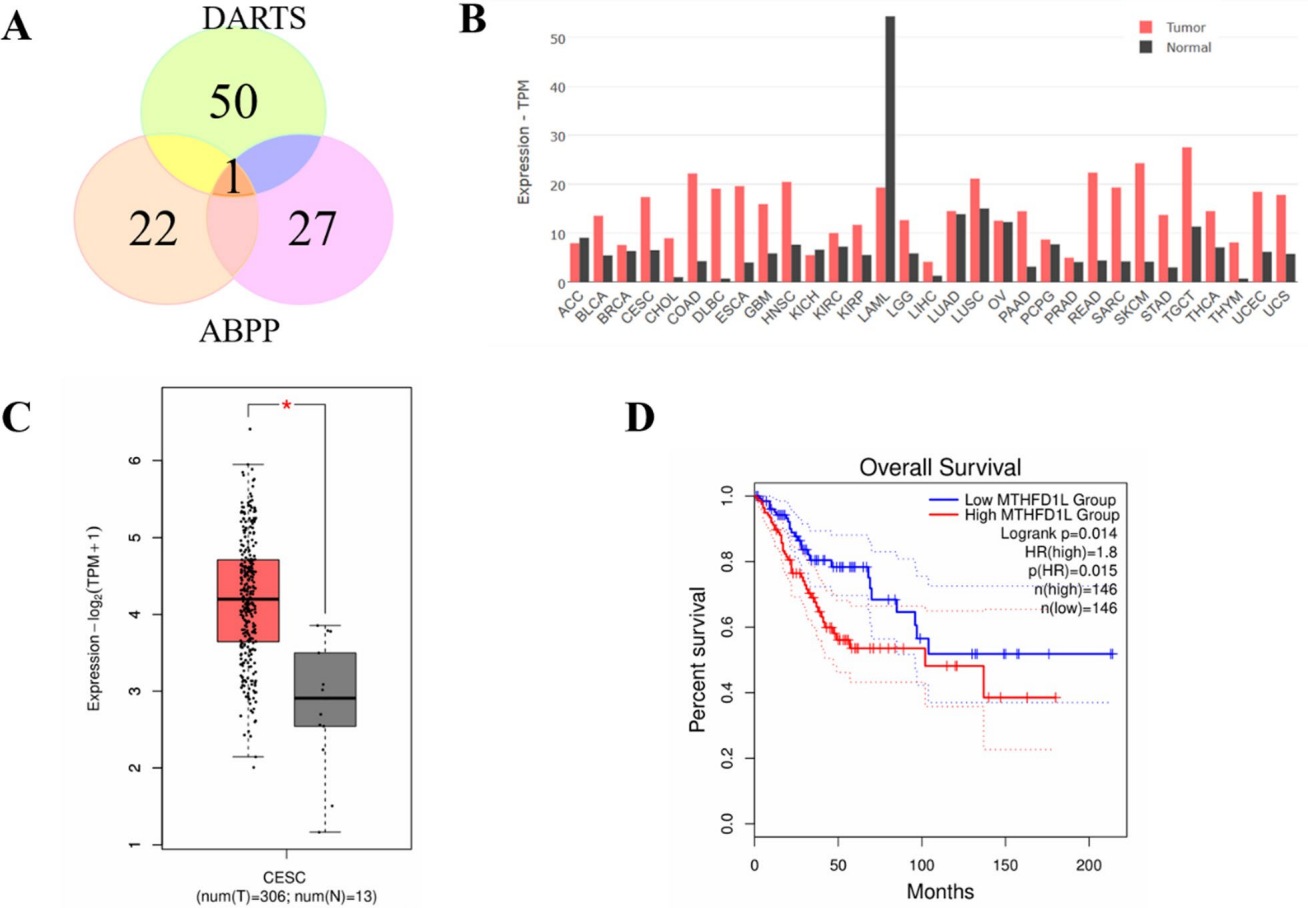
MTHFD1L is one of the most potential binding partners of PAA and highly involved with cancer through bioinformatic analysis. **A** Vanne group demonstrating the potential binding partners of PAA identified by DART and ABPP strategies. **B** The gene expression profile of MTHFD1L across all tumor lines and paired normal tissues. The height of bar stands for the median expression of certain tumor type or normal tissue. **C** The expression of MTHFD1L in CESC was analyzed through GEPIA2 database. The expressions of MTHFD1L in CESC patients (n = 306) and normal people (n = 13) in the TCGA database were analyzed. **D** The overall survival analysis of MTHFD1L in CESC cancer cell line through TCGA database showed the significant prognostic impact of MTHFD1L

Among all potential binding targets of PAA detected by two different strategies, methylenetetrahydrofolate dehydrogenase 1 like (MTHFD1L) protein is the only overlapped target (Fig. 3A). We therefore propose MTHFD1L is the most potential binding target of PAA. MTHFD1L is the human mitochondrial C1-terrahydrofolate synthase, catalyzing the conversion of tetrahydrofolate (THF) to 10-formyltetrahydrofolate (10-CHO-THF) in mitochondria [16, 17]. It is the core enzyme in the one-carbon (1C) cycle metabolism, which is highly expressed in a variety of tumors and associated with tumor cell proliferation and invasion [18, 19].

To get a deeper understanding of gene MTHFD1L functions in tumor cells, we further mined the data through The Cancer Genome Atlas (TCGA) databases (https://www.cancer.gov/tcga) and Genotype-Tissue Expression (GTEx) database [20]. Clinical data from two databases demonstrated that MTHFD1L is differentially overexpressed in multiple cancer subtypes and paired normal tissues (Fig. 3B). Since PAA exhibited decent antitumor activity in HeLa cell (cervical cancer cell line), we paid extra attention to the expression profiles of MTHFD1L in cervical &endocervical cancer (CESC) line. Gene expression profile showed that MTHFD1L is highly overexpressed in CESC patients, compared to the expression in normal people (Fig. 3C). Furthermore, survival analysis allows the identification of correlation between gene expression and prognostic outcomes. We performed the survival analysis to evaluate clinical relevance of MTHFD1L gene through GEPIA2 [21]. Result was shown in Fig. 3D and we found that MTHFD1L exhibited significant association with unfavorable prognostic outcome in CESC cancer, but not in other cancers (Figure S2). Taken together, comprehensive bioinformatic analysis has aided the understanding of the important role of MTHFD1L gene in CESC cancer type and led to the identification of MTHFD1L as a potential therapeutic target and biomarker.

### Verification of the direct interaction between MTHFD1L and PAA

We used nuclear magnetic resonance (NMR) saturation transfer difference (STD) and surface plasmon resonance (SPR) techniques to verify the direct interaction between MTHFD1L and PAA in the molecular level.

**Fig. 4:**
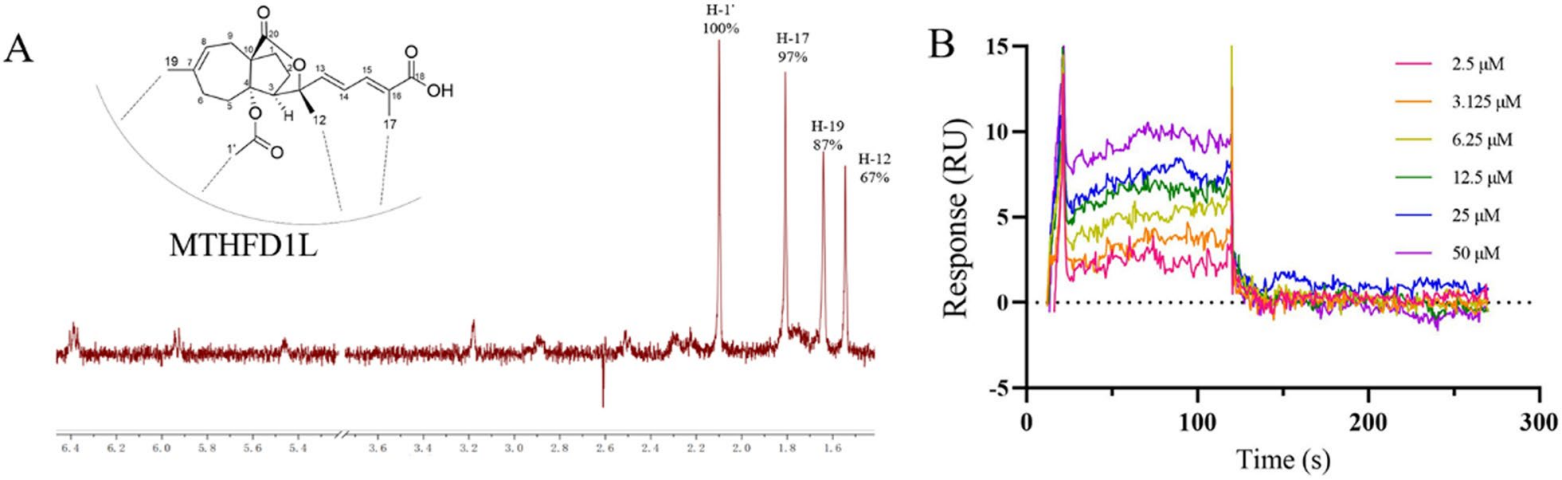
PAA directly interacts with MTHFD1L. **A** Chemical structure of PAA and its relative orientation with MTHFD1L revealed by NMR STD assay, with the ^1^H STD spectrum as red. **B** SPR assay showing the direct interaction between PAA and MTHFD1L. The steady state analysis method was used to fit the dissociation constant

The ligand-observed NMR STD studies could determine the direct protein–ligand interaction and provide information on the putative orientation of ligand to target protein. Protons in the PAA closest to the protein upon binding show the strongest STD effect. The STD effect of PAA is shown in Fig. 4A. H-1’ gives the strongest enhancement and is considered to interact the most with the protein surface. This observation is consistent with the previous structure–activity relationship (SAR) analysis that H-1’ is essential for the activity of PAA. Both our previous data and reports from other groups have demonstrated that deacetyl derivatives of PAA exhibit no antitumor activity, indicating that the C4-acetoxy group is crucial for this activity [10, 22]. Notably, H-1’ is located within the C4-acetoxy group. Pseudolaric acid C (PAC), which lacks the -COOCH3 moiety and possesses a C4 hydroxyl group instead, is also inactive. H-17 and H-19 exhibited similar but slightly less STD effect, indicating the critical importance of these two groups in the MTHFD1L-PAA binding mode, in accordance with the SAR result. STD effects of H-12 and other protons suggest that these protons are not in direct contact with the protein. Taken together, NMR STD method demonstrated PAA directly interacts with target protein MTHFD1L in a defined orientation that the -OAc moiety and H-17 closely faces the protein surface.

We further verified and quantify the binding affinity of compound PAA to the target protein MTHFD1L through surface plasmon resonance (SPR) binding experiments (Fig. 4B). The equilibrium dissociation constant (Kd) is obtained by steady-state affinity analysis. Compound PAA and target protein MTHFD1L are fast binding and fast dissociation binding modes, and the equilibrium dissociation constant K_d_ is fitted, with K_d_ about 4.05 μM. The SPR results further confirmed the direct interaction between PAA and MTHFD1L.

### Molecular docking analysis to generate a possible binding mode of the PAA to MTHFD1L

To gain the insight into the possible binding mechanism between PAA and MTHFD1L, molecular docking analysis with Autodock was performed. Due to the lack of published crystal structure, we adopted AlphaFold2 to predict the three-dimensional structure of MTHFD1L protein. The ligand with binding energies lower than − 7 kcal/mol was identifies as potential binding. The docking studies showed that PAA was well located within the predicted ATP—binding pocket of the MTHFD1L, with the binding energy of − 7.88 kcal/mol. This model with the lowest energy was chosen for subsequent protein–ligand interface analysis.

**Fig. 5:**
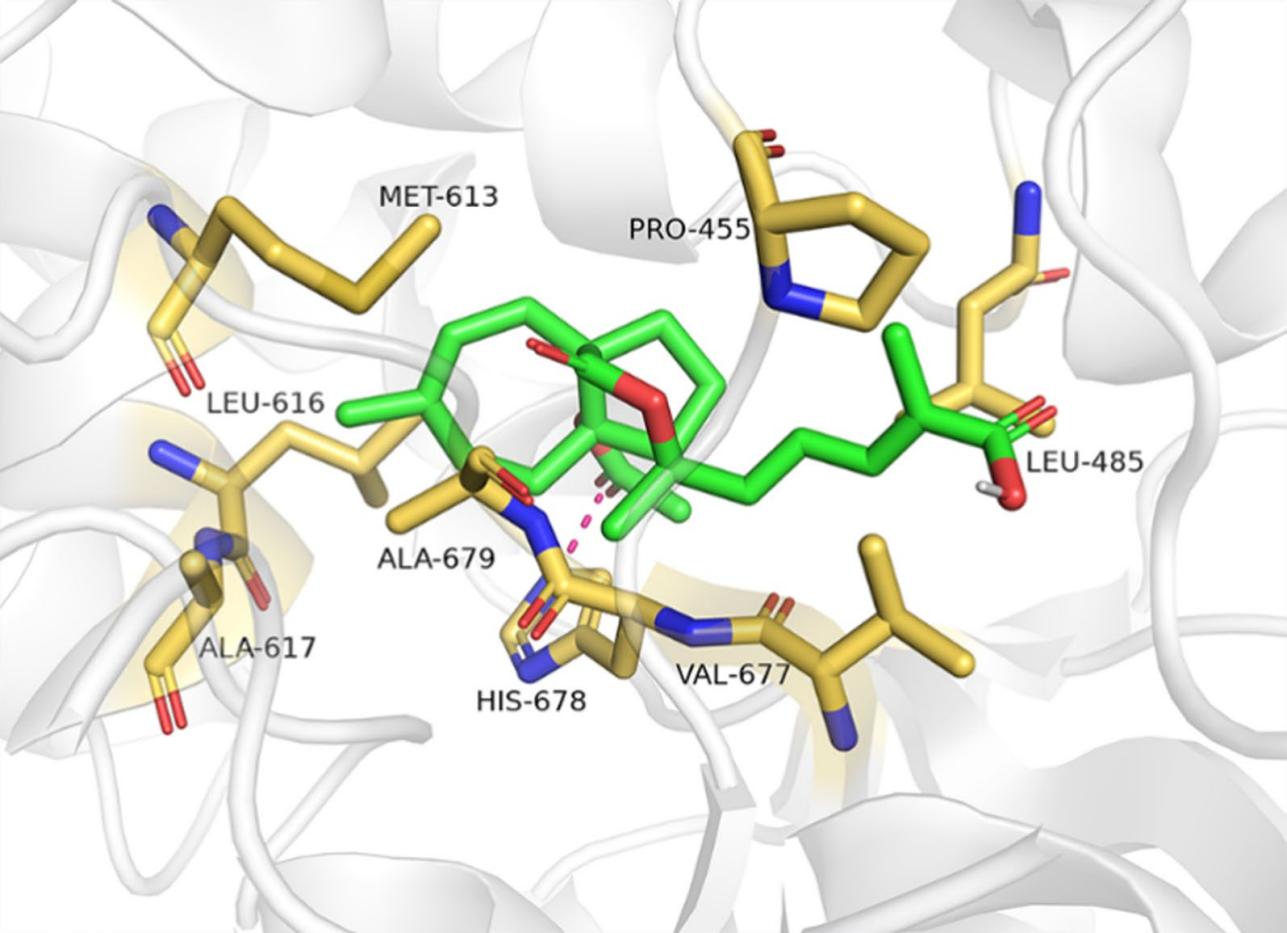
Molecular docking analysis exhibits the binding interactions between PAA and MTHFD1L. Ligand PAA is denoted in green. The hydrophobic residues of MTHFD1L are colored in yellow

As shown in Fig. 5, H-1’ and H-19 are embedded in the center of the ATP binding pocket of MTHFD1L, suggesting its potential inhibition mechanism for MTHFD1L. C-19 forms hydrophobic interaction with residues M613, L616 and A617. The carbonyl group at C-4 (C-1’ group) forms hydrogen bond with the side chains of H678. C-17 methyl group forms interactions with the hydrophobic pocket formed by residues L485, P455 and V677 of MTHFD1L. Taken together, the hydrophobic interactions between protons in the PAA (C-1’, C-17 and C-19) and MTHFD1L strengthen the position of PAA in the ATP binding pocket of MTHFD1L. The groups of PAA highly involved in the interaction with MTHFD1L from molecular docking results are in line with the gathered experimental NMR STD data.

### Accumulation of reactive oxygen species (ROS) mediates the antitumor activity

**Fig. 6:**
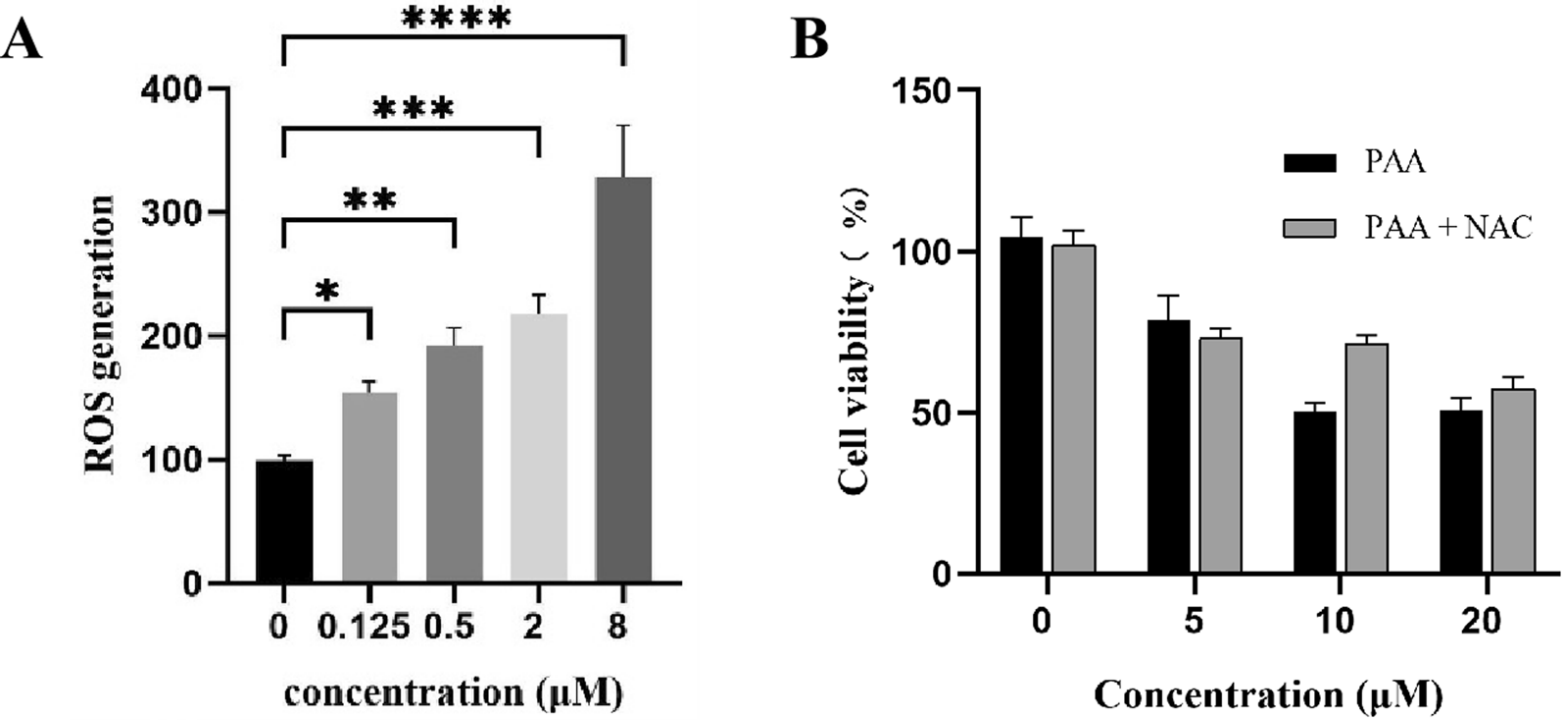
**A** Generation of ROS after treatment with different concentrations of PAA in HeLa cells. **B** Cell viability after treatment with different concentrations of PAA in the absence or presence of ROS scavenger NAC

The accumulation of ROS, the metabolite products of mitochondria, is strongly associated with the regulation of tumor cell death [23]. Elevated levels of ROS can cause irreversible damage to cellular components, leading to apoptosis and cell death. ROS overproduction serves as an important mediator of cell death in response to various stimuli. Li Hao et al. have reported that knockdown of MTHFD1L increased ROS levels and subsequently accelerated tumor cell death under oxidative stress conditions [19]. We sought to investigate the consequences of MTHFD1L inhibition by PAA in HeLa cells. The ROS level were measured in both untreated and PAA-treated HeLa cells, with the level in control group normalized to 100%. Results demonstrated a concentration-dependent increase in ROS levels upon PAA treatment (Fig. 6A).

To further elucidate the role of ROS production in PAA-induced tumor cell death, we employed the ROS scavenger N-acetylcysteine (NAC) to assess its impact on HeLa cell viability. Our results demonstrated that pre-treatment of HeLa cells with NAC (5 mM) significantly restored cell viability across various concentrations of PAA (Fig. 6B). This finding indicates that the antitumor activity of PAA is compromised by the ROS inhibitor NAC. These data suggest that PAA stimulates the accumulation of ROS, which serves as a critical mediator of PAA-induced tumor cell death.

### Antitumor activity of MTHFD1L in HeLa cells

The MTHFD1L knockdown assay was performed to explore the functional relevance of MTHFD1L in the antitumor activity. HeLa cells were transfected with different siRNAs against MTHFD1L.The knockdown efficiency was assessed using quantitative real-time polymerase chain reaction (qRT-PCR) (Figure S3A). Our results demonstrated that cell viability was significantly reduced following MTHFD1L knockdown in HeLa cells (Figure S3B), consistent with previous reports showing that siRNA-mediated knockdown of MTHFD1L inhibits cell proliferation in colorectal cancer and papillary thyroid cancer [18, 24]. This observation limits our ability to evaluate the antitumor activity of PAA in the MTHFD1L knockdown cells.

### Integrated transcriptome and network pharmacology analysis

**Fig. 7:**
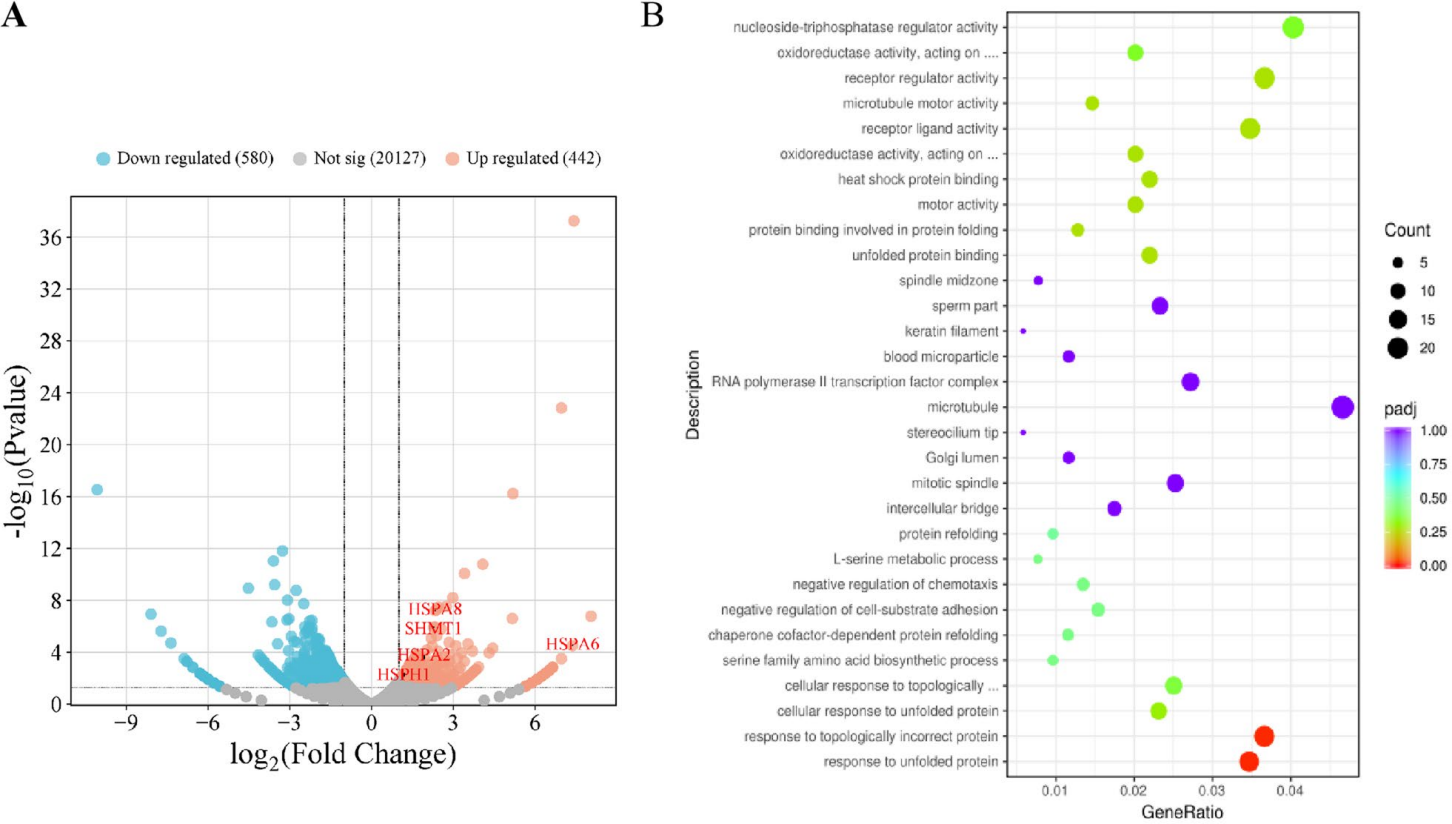
Transcriptome analysis of HeLa cells after treatment with PAA. **A** Volcano plot of differential gene expression associated with PAA treatment, with the horizontal axis showing the gene fold change and vertical axis indicating the *p* value. The red and blue circles indicate the up- and down- regulated genes, respectively. **B** Functional categorization of up- and down- regulated genes based on the Gene ontology (GO) annotations

We used the RNA sequencing to assess the overall effect of PAA on the gene expressions in HeLa cells. Analysis (Fig. 7A) revealed that 442 genes were up-regulated and 580 genes were down-regulated, respectively (Table S3). Gene ontology (GO) biological process analysis and Kyoto Encyclopedia of Genes and Genomes (KEGG) enrichment analysis showed that PAA affected a wide range of biological functions, mainly including protein folding/refolding, response to unfolded protein/topologically incorrect protein, heat shock protein binding, providing evidences that PAA potentially targets multiple targets, including heat shock protein 90 (Fig. 7B). This is consistent with our previous report that Hsp90 is one of the main binding targets of PAA. The biosynthesis of amino acids, tyrosine metabolism, glycine, serine and threonine metabolisms were the significantly enriched pathways, supporting that PAA regulates the 1C unit metabolism.

## Conclusion

In this study, we comprehensively integrated labeled ABPP strategy and label-free DARTS techniques to identify the potential target of PAA. We demonstrate that MTHFD1L is one of protein targets in HeLa cells. The direct interaction between MTHFD1L and PAA was further verified. The binding constant was fitted and calculated. The potential binding mode of MTHFD1L-PAA interaction was analyzed and further demonstrated by NMR STD and SPR techniques. This study utilized comprehensive investigations to identify MTHFD1L as one of the effective targets of PAA.

MTHFD1L catalyzes the last step in the flow of 1C units from mitochondria to cytoplasm [25]. Other key enzymes regulating the folate pathway and serving as critical role in one-carbon metabolism reactions, include MTHFD2 and serine hydroxymethyl transferase 1 (SHMT1). We constructed a string pathway network for these proteins. We paid attention to the genes in the folate pathway and 1C unit metabolism (Figure S4). Results showed that the expression of SHMT1 was up-regulated after PAA treatment, indicating the activation of the formate synthesis in cytoplasm and the inhibition of synthesis pathway in the mitochondria, which further supports that PAA treatment inhibited the function of MTHFD1L in mitochondria. On the other hand, the observation of up-regulation of different isoforms of heat shock protein HSP70, including HSPA2, HSPA6 and HSPA8, is consistent with our previous data that PAA treatment promotes the compensatory overexpression of HSP70 protein. The observation of the interaction network analysis aligns with the discovery of protein targets.

However, we could not rule out the possibility that other targets may involve in the anticancer activity of PAA. Accumulating evidences showed that natural products interact with multiple protein targets rather than single target. Further attention and effort need to be paid to the broad-spectrum targets of PAA.

## Experimental section

### Extraction and isolation of PAA

PAA employed in this research was extracted from *Pseudolarix cortex* following the procedures described previously [10]. The ^1^H and ^13^C NMR spectra of compound PAA was shown in supplementary information (Figure S1).

### Cell counting kit-8 (CCK8) assay

Individual cell suspensions are prepared with culture medium (DMEM) supplemented with fetal bovine serum, penicillin and streptomycin, and then inoculated into 96-well plates at 1 × 10^4^ cells per well. Cells were incubated at 37 °C to grow till about 70%. Compounds were added to cells at concentrations of 40, 8, 1.6, 0.32 and 0.064 μM, respectively, and incubated for 24 h. There were three replicate wells for each treatment. Lastly, CCK-8 solution was added and incubated for 1 h. The wavelength was set to 450 nm, and each well’s light absorption value was read by multifunctional enzyme standard (Flex station 3). The results were recorded and the cell growth curve was plotted. The IC_50_ values of the compounds were calculated by PRISM 8.0.1.

### Pull-down assay

HeLa cells were grown in DMEM, collected, lysed on ice for 10 min and then centrifuged. Protein concentrations of supernatant were assessed by BCA assay kit from Beyotime, China. Three groups were divided as follows: control group, PAA probe group (10 μM), competition group (10 μM PAA probe + 500 μM PAA). Following operations followed standard protocol. The readout is visualized by Amersham Typhoon Biomolecular Imager in-gel fluorescence scanning at 533 nm. The visualized bands with intensity difference between PAA probe group and control group were cut into pieces and subjected to mass spectrometry to investigate the potential binding proteins targets of PAA.

### DARTS assay

A dish of 100 mm^2^ HeLa cells was washed three times with pre-cooled PBS 500 μL of M-per lysis solution and 5 μL of protease inhibitor were added into the dish. Cells were lysed and then centrifuged. The protein concentration was detected by the BCA assay kit. 200 μL of each cell lysate was divided into two groups. 300 μg protein were incubated with 1 μL 20 mM (100 μM final concentration) PAA, for 1 h in a metal bath at 25 °C. The pronase was prepared into 10 mg/ml stock solution. Samples were treated with pronase with different concentrations. The ratios of pronase to protein samples were 1:0, 1:100, 1:200, 1:400, 1:1000 and 1:2000 (w/w), respectively, for the preliminary enzyme optimization. The final ratio of pronase to protein sample used for the mass spectrometry analysis is 1:100. After incubation for 25 min at room temperature, the reaction was terminated by boiling in SDS-PAGE loading buffer. The sample proteosome was separated via SDS-PAGE and stained with Coomassie blue. The differential bands corresponding to PAA protected proteins towards the proteolysis were observed and cut into pieces, which were subsequently analyzed through mass spectrometry analysis. The quantification and sequencing analysis were performed by Novogene.

### Molecular docking analysis

The predicted protein structure of MTHFD1L (Q3V3R1) was sourced from Alphafold (https://alphafold.ebi.ac.uk/), and the prediction of ligands binding pocket is processed by Prankweb (https://prankweb.cz/). The Autodock Tools were applied for molecular docking studies following the standard protocol.

### MTHFD1L cDNA cloning and construction

The cloning and expression of the recombinant fusion protein with maltose binding protein (MBP) were following the protocols descried previously with minor modification [16]. The DNA fragment encoding the MTHFD1L (68–978 AA) was PCR-amplified and cloned into the pMAL-c5x vector with the NotI and BamHI sites. The cloning yields an N-terminally His_10_-tagged protein with a thrombin cleave (LVPRGS) site between His_10_-tag and maltose binding protein (MBP) tag. The sequence of the complete final construct MBP-MTHFD1L was confirmed by the sequencing.

### Over-expression and purification of MTHFD1L protein

Recombinant MBP-MTHFD1L construct was transfected and overexpressed in the *E. coli* BL21 (DE3) strain. The cultures were grown in LB medium at 37 °C until the O.D._600_ value reached 0.4, and then induced with 10 μM isopropyl β-d-1-thiogalactopyranoside (IPTG) at 4 °C for 4 h. The cells were harvested by centrifugation, resuspended in lysis buffer with PMSF and were sonicated on ice. To purify the MBP-MTHFD1L fused protein, cell extracts were purified by Ni–NTA (5 mL, GE Healthcare) affinity chromatography. The eluted recombinant protein was further digested with thrombin (1:300) for 4 h. After MBP cleavage, the MTHFD1L protein solution was further purified using size-exclusion chromatography (Superdex 75, GE Healthcare). The purity of the final recombinant MTHFD1L protein was higher than 90% based on the sodium dodecyl sulfate–polyacrylamide gel for electrophoresis (SDS-PAGE) after Coomassie blue staining.

### ROS detection

The production of ROS was detected by Reactive Oxygen Species Assay Kit. In the cells, the fluorescent probe 2’,7’-dichlorodihydrofluorescein diacetate (DCFH-DA) was hydrolyzed by esterase to generate DCFH, which is oxidized by ROS to produce fluorescent 2’,7’-dichlorofluorescein (DCF). The ROS level is quantified through the fluorescence intensity of DCF. After treatment with PAA, PAA-probe for 3 h, HeLa cells were washed three times and incubated with DCFH-DA (10 μM) in the dark for 30 min. Cells were washed twice with cold serum-free DMEM. The fluorescence intensity was measured in a spectrophotometer at an excitation wavelength of 485 nm and an emission wavelength of 520 nm.

### NMR STD assay

The molecular interaction of PAA binding to MTHFD1L was studied by saturation transfer difference (STD) NMR experiments. The STD experiment was performed by a Bruker Fourier spectrometer (800 MHz). The NMR buffer was PBS buffer in 90% D_2_O and 2.5% DMSO-*d*_6_. NMR STD sample was prepared by 1 mM PAA in the presence or absence of recombinant MTHFD1L protein. On-resonance irradiation of protein was performed at 0.5 ppm, with off-resonance was set at 40 ppm. The decrease of the signal intensity of the ^1^H spectrum indicated the saturation transfer and the interaction between protein and ligand. The equation STD = (*I*_o_–*I*_sat_)/*I*_o_ was utilized to quantify the STD effect, while *I*_o_ is the off-resonance saturation, and *I*_sat_ is the on-resonance saturation. Software TopSpin (Bruker Corporation) was used for data processing.

### Surface plasmon resonance analyse

SPR examinations were conducted utilizing a Biacore T200 (Cytiva) equipped with CM5 research-grade sensor chips (Cytiva). 100 μg /mL of recombinant MTHFD1L protein were immobilized on CM-5 sensor chip using standard amine-coupling protocols. Compound PAA was prepared in running buffer at different concentrations, including 2.5, 3.125, 6.25, 12.5, 25, 50 and 100 μM, respectively and injected on to the chip. Binding experiments were conducted at 25 °C, with monitoring of association for 120 s and dissociation for 180 s. The response unit (RU) signal was calculated through subtracting the reference response. A set of sensorgram at different concentrations of PAA were obtained. The equilibrium dissociation constant (K_D_ value) of protein–ligand were fitted by affinity fitting the data to steady-state model (Biacore, Sweden).

### Transcriptome analysis

Cells were pretreated with PAA at 2 μM for 24 h and then collected to extract the total RNA. We used Qubit RNA BR (Broad-Range) assay kit to determine the concentration of the RNA and RNA Nano 6000 assay kit with Bioanalyzer system 2100 (Agilent technologies) to check the integrity of RNA. An illumine HiSeq X Ten sequencer was utilized to conduct RNA-seq. FastQC tool was conducted to perform the quality control of the raw data. Differential expression analysis of two groups (control group and PAA-treated group) were performed as described previously [26]. The criteria to identify genes of interest were fold change with the expression of │log_2_ fold change (FC)│ > 1 and *p*-value (*p* value < 0.05). Any genes, which met these criteria, were either up-regulated or down-regulated genes. They were identified as differentially expressed genes (DEG) and selected for further investigation.

### Bioinformatic analysis

MTHFD1L expression data of cervical squamous cell carcinoma and endocervical adenocarcinoma (CESC) was obtained from the TCGA databases and analyzed through the Gene Expression Profiling Interaction Analysis (GEPIA2), an outstanding web tool to analyze the gene expression data of tumors and normal samples [27]. The interaction network was developed using Cytoscape [28].

### RNA interference and cell viability assay for MTHFD1L knockdown cells

HeLa cells were transfected with siRNA purchased from Sangon Biotech (Shanghai). Transfection was conducted using Lipofectamine RNAiMAX (Life Technologies) according to the instructions provided in the kit manual. Quantitative real-time polymerase chain reaction (qRT-PCR) was used to measure the target knockdown efficiency. Standard CCK8 assay was used to evaluate the cell viability. The absorption was recorded by multifunctional enzyme standard (Flex station 3) at 450 nm.

## Supplementary Information

Additional file 1.

## Data Availability

Data will be made available on reasonable request.
