# Supplementary material for: Identification and verification of methylenetetrahydrofolate dehydrogenase 1-like protein as the binding target of natural product pseudolaric acid A
Source: Nat Prod Bioprospect. 2025 Apr 2;15(1):21. doi: 10.1007/s13659-025-00502-1 (PMC11965064; doi:10.1007/s13659-025-00502-1)
Supplement: Supplementary file 1 — Additional file 1. [file 13659_2025_502_MOESM1_ESM.docx]

**Identification and verification of** **methylenetetrahydrofolate dehydrogenase 1-like protein as the binding target of natural product pseudolaric acid A**

Haoqi Dong ^1, 2†^ , Xinni Yang ^1†^, Peiying Wang ^2,3^, Weiya Huang ^2,3^, Liang Zhang ^1^,

Song Song^4^, Jiangxin Liu ^1*^

^1^ Key Laboratory of Phytochemistry and Natural Medicines, Kunming Institute of Botany, Chinese Academy of Sciences, Kunming, 650201, China

^2^ University of Chinese Academy of Sciences, Beijing, 100049, China

^3^ Yunnan University, Kunming, 650500, China

^4^ State Key Laboratory of Natural and Biomimetic Drugs, School of Pharmaceutical Sciences, Peking University, Beijing 100191, China

^†^ These authors contributed equally to this work

* Corresponding author. E-mail: liujiangxin@mail.kib.ac.cn (J. LIU);

**Figure S1.** ^1^H and ^13^C NMR spectra of PAA……………………………………………………2

**Figure S2.** The overall survival analyses based on the cancer type and cancer subtypes……..3

**Figure S3.** MTHFD1L knockdown inhibits the cell viability of HeLa cells ………………….4

**Figure S4.** Interaction network of PAA and differential expressed genes …………………….5

**Table S1** List of PAA putative binding partner proteins, identified by DARTS approach….....6

**Table S2** List of PAA putative binding partner proteins, identified by ABPP approach…........8

**Table S3** Transcriptome analysis revealed the up-regulated genes and down-regulated genes………………………………….…………………………………………….. ………...9

**Figure S1.** ^1^H and ^13^C NMR spectra of compound PAA.

**
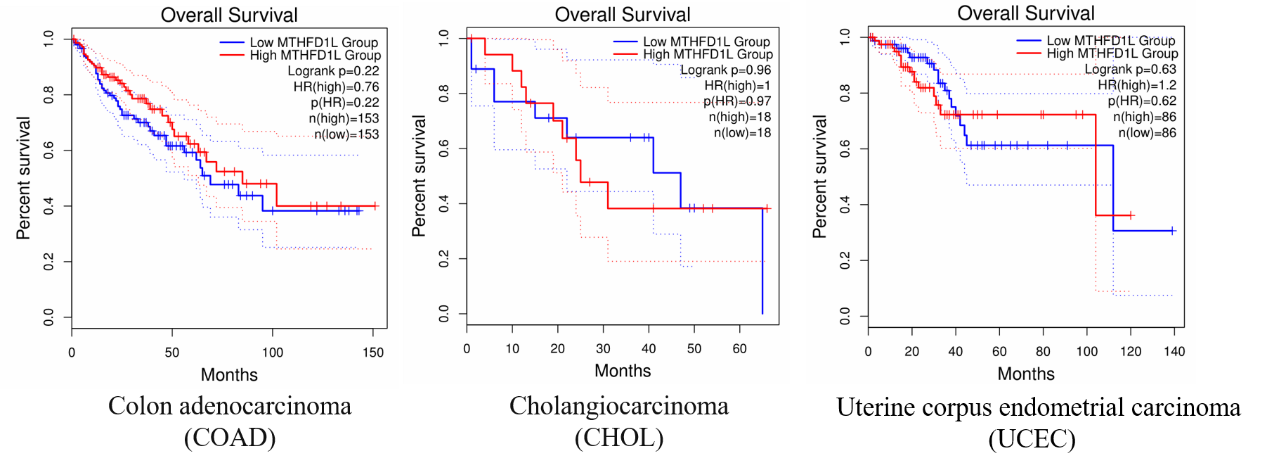
**

**Figure S2.** The overall survival analyses based on the cancer type and cancer subtypes showed the non-significant prognostic impact of MTHFD1L in other cancer types.


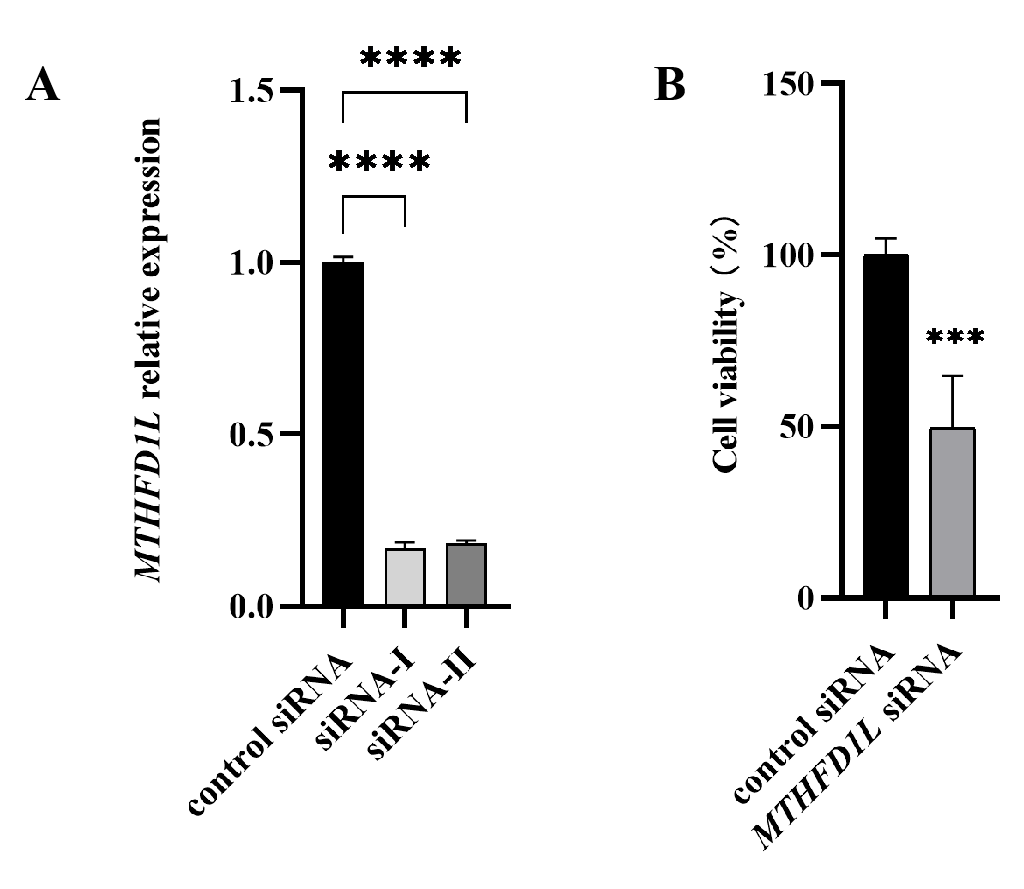


**Figure S3**. A. The relative expressions of MTHFD1L in HeLa cells after transfection with control or MTHFD1L siRNAs. B. Cell viability assay of HeLa cells after transfection with siRNA.


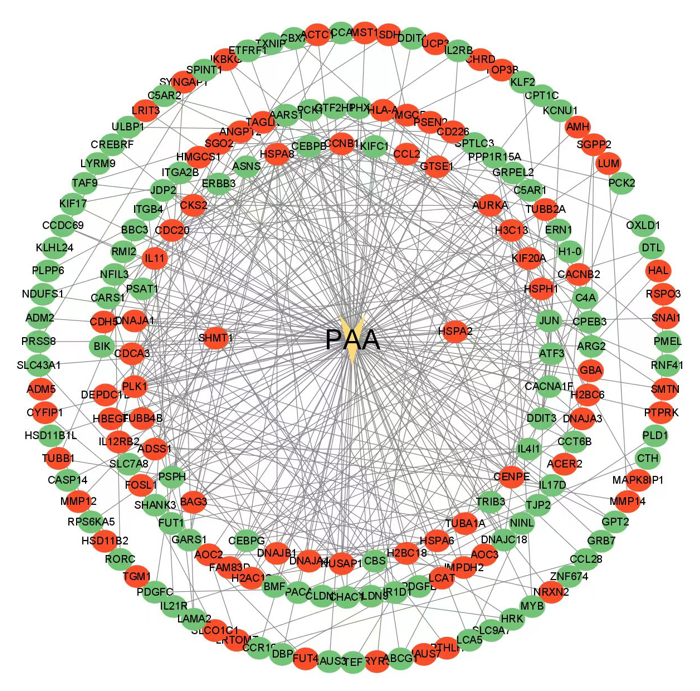


**Figure S4.** Interaction network of PAA and differential expressed genes, with up-regulated genes colored red and down-regulated genes colored green, constructed and visualized by Cytoscape software.

**Table S1**. List of PAA putative binding partner proteins, identified by DARTS approach.

For each reported protein, the following parameters are presented: Uniprot accession No., molecular weight (Mass), gene name, fold change score and significance.

| Uniprot accession No. | Molecular weight (Mass/Da) | Gene | Fold change score | Significant |
| --- | --- | --- | --- | --- |
| Q9Y490 | 269767 | TLN1 | 1.832071771 | Significant |
| Q5D862 | 248073 | FLG2 | 2.162869211 | Significant |
| A0A0A0MTH9 | 206887 | BTAF1 | 1.767608235 | Significant |
| Q9UBG0 | 166674 | MRC2 | 2.881107081 | Significant |
| O75694 | 155199 | NUP155 | 1.691780062 | Significant |
| C9J5X1 | 154791 | IGF1R | 1.739593763 | Significant |
| O95487 | 137418 | SEC24B | 2.94013125 | Significant |
| D3DTH7 | 121682 | MYO1C | 1.630542086 | Significant |
| G5EA31 | 111985 | SEC24C | 2.863081689 | Significant |
| A0A6Q8KRG2 | 111658 | GOLGA2 | 2.015216752 | Significant |
| Q96CW5 | 103571 | TUBGCP3 | 1.91057204 | Significant |
| Q9BSJ2 | 102534 | TUBGCP2 | 1.714941826 | Significant |
| H0Y938 | 101533 | COPB2 | 2.442434278 | Significant |
| A0A087WVM4 | 99312 | MTHFD1L | 1.739481953 | Significant |
| Q12979 | 97598 | ABR | 3.685916886 | Significant |
| A0A7I2V454 | 94203 | NEK9 | 1.695643135 | Significant |
| A0A024R179 | 91839 | NCBP1 | 1.961266275 | Significant |
| Q93034 | 90955 | CUL5 | 1.790865172 | Significant |
| A0A090N7U0 | 89679 | CUL1 | 1.93808844 | Significant |
| Q01813 | 85596 | PFKP | 1.934479307 | Significant |
| O60645 | 85567 | EXOC3 | 2.003914611 | Significant |
| P43304 | 80853 | GPD2 | 1.632518249 | Significant |
| Q12931 | 80110 | TRAP1 | 2.612487912 | Significant |
| P54136 | 75379 | RARS1 | 1.632800355 | Significant |
| O43390 | 70943 | HNRNPR | 1.874169214 | Significant |
| V9HW22 | 70898 | HEL-S-72p | 1.847810141 | Significant |
| Q6IB91 | 70697 | PCK2 | 1.963194692 | Significant |
| H6VRF8 | 66053 | KRT1 | 1.79236525 | Significant |
| P35908 | 65433 | KRT2 | 1.768785094 | Significant |
| Q96AY3 | 64245 | FKBP10 | 3.154053818 | Significant |
| Q9NZI8 | 63481 | IGF2BP1 | 12.79462735 | Significant |
| A0A1B0GVI3 | 63346 | KRT10 | 1.964909494 | Significant |
| A0A0U1RQT1 | 62842 | ACAP2 | 3.403527011 | Significant |
| A0A0S2Z428 | 60045 | KRT6A | 7.154918461 | Significant |
| Q15758 | 56598 | SLC1A5 | 2.143539031 | Significant |
| P11169 | 53924 | SLC2A3 | 1.988266781 | Significant |
| Q8WUN4 | 51682 | FAM40A | 1.791551245 | Significant |
| Q05639 | 50470 | EEF1A2 | 1.968769017 | Significant |
| B4DEX8 | 39710 | MAT2A | 1.961273458 | Significant |
| B8ZZQ4 | 21076 | PTCD3 | 1.681492887 | Significant |
| E5RGY0 | 17221 | DERL1 | 3.899438635 | Significant |
| P12273 | 16572 | PIP | 1.748940387 | Significant |

Table S2. List of PAA putative binding partner proteins, identified by ABPP approach.

For each reported protein, the following parameters are presented: Uniprot accession No., molecular weight (Mass), gene name, fold change score and significance.

| Uniprot accession No. | Molecular weight (Mass/Da) | Gene | Fold change score | Significant |
| --- | --- | --- | --- | --- |
| A0A2P9DU05 | 160900 | ROCK2 | 30.58294894 | significant |
| Q9NVI1 | 149324 | FANCI | 4.358974585 | significant |
| Q12768 | 134286 | WASHC5 | 16.00930309 | significant |
| H7BXY3 | 130550 | DHX30 | 45.42838134 | significant |
| O60518 | 124714 | RANBP6 | 22.13907091 | significant |
| A0A024R7L5 | 123036 | UPF1 | 7.919359933 | significant |
| A0A024R3R6 | 110516 | NUP133 | 3.589840414 | significant |
| A0A3B3IT92 | 100736 | MCM4 | 5.528665692 | significant |
| A0A087WVM4 | 99312 | MTHFD1L | 8.451963294 | significant |
| Q6IA86 | 92500 | ELP2 | 3.654084165 | significant |
| P48960 | 91869 | ADGRE5 | 3.567607426 | significant |
| A0A7P0T9L3 | 90391 | CUL4B | 7.976139531 | significant |
| V9HWB4 | 72333 | HEL-S-89n | 4.080331267 | significant |
| C9IZ01 | 66035 | GFM1 | 8.421724132 | significant |
| V9HWH7 | 64616 | HEL-S-70p | 51.84979736 | significant |
| A0A024R1U0 | 63542 | RANGAP1 | 7.682589922 | significant |
| P30740 | 42742 | SERPINB1 | 4.533441081 | significant |
| A0A7P0TB04 | 28919 | BSG | 7.125654225 | significant |

Table S3. Transcriptome analysis revealed the up-regulated genes and down-regulated genes. The identified novel genes were not listed.

| Uniprot accession No. | Gene | Log_2_FoldChange | UP |
| --- | --- | --- | --- |
| ENSG00000114942 | EEF1B2 | 2.467110289 | up |
| ENSG00000109971 | HSPA8 | 2.331911987 | up |
| ENSG00000167552 | TUBA1A | 2.324277917 | up |
| ENSG00000176974 | SHMT1 | 2.273723152 | up |
| ENSG00000129646 | QRICH2 | 2.399438057 | up |
| ENSG00000144559 | TAMM41 | 2.190557774 | up |
| ENSG00000126562 | WNK4 | 2.845560505 | up |
| ENSG00000173110 | HSPA6 | 7.373285137 | up |
| ENSG00000163082 | SGPP2 | 3.087274746 | up |
| ENSG00000280213 | UCKL1-AS1 | 4.440604456 | up |
| ENSG00000273749 | CYFIP1 | 2.032213111 | up |
| ENSG00000215630 | GUSBP9 | 3.701261313 | up |
| ENSG00000177628 | GBA | 2.44378793 | up |
| ENSG00000126231 | PROZ | 4.310080932 | up |
| ENSG00000182685 | BRICD5 | 3.302505239 | up |
| ENSG00000185100 | ADSS1 | 1.684392943 | up |
| ENSG00000274615 | NPEPPSP1 | 2.859735481 | up |
| ENSG00000183963 | SMTN | 1.613458093 | up |
| ENSG00000139146 | SINHCAF | 1.76295741 | up |
| ENSG00000174358 | SLC6A19 | 1.694045457 | up |
| ENSG00000262406 | MMP12 | 1.743254115 | up |
| ENSG00000278311 | GGNBP2 | 1.800075325 | up |
| ENSG00000188818 | ZDHHC11 | 1.577962367 | up |
| ENSG00000266235 | MIR3176 | 3.145657521 | up |
| ENSG00000197283 | SYNGAP1 | 6.961145229 | up |
| ENSG00000126803 | HSPA2 | 1.929573683 | up |
| ENSG00000104899 | AMH | 1.745845808 | up |
| ENSG00000159251 | ACTC1 | 2.226108108 | up |
| ENSG00000101447 | FAM83D | 1.459609589 | up |
| ENSG00000186567 | CEACAM19 | 1.727519997 | up |
| ENSG00000112972 | HMGCS1 | 1.442463475 | up |
| ENSG00000267745 | ENSG00000267745 | 2.638173683 | up |
| ENSG00000279602 | ENSG00000279602 | 2.638173683 | up |
| ENSG00000110172 | CHORDC1 | 1.575470912 | up |
| ENSG00000112984 | KIF20A | 1.407057427 | up |
| ENSG00000087586 | AURKA | 1.394424823 | up |
| ENSG00000157045 | NTAN1 | 2.173997115 | up |
| ENSG00000086061 | DNAJA1 | 1.376177051 | up |
| ENSG00000092295 | TGM1 | 2.4283012 | up |
| ENSG00000171428 | NAT1 | 2.4283012 | up |
| ENSG00000177943 | MAMDC4 | 1.813641817 | up |
| ENSG00000290796 | ENSG00000290796 | 2.905867598 | up |
| ENSG00000232546 | ENSG00000232546 | 3.920351665 | up |
| ENSG00000205085 | GARIN1A | 2.300098084 | up |
| ENSG00000084110 | HAL | 6.642108917 | up |
| ENSG00000248367 | ENSG00000248367 | 6.642108917 | up |
| ENSG00000265806 | MIR4292 | 6.642108917 | up |
| ENSG00000203772 | SPRN | 2.381183004 | up |
| ENSG00000255198 | SNHG9 | 3.251216449 | up |
| ENSG00000146374 | RSPO3 | 2.630607743 | up |
| ENSG00000166851 | PLK1 | 1.294612102 | up |
| ENSG00000258441 | LINC00641 | 1.444928455 | up |
| ENSG00000137496 | IL18BP | 1.4753703 | up |
| ENSG00000131480 | AOC2 | 1.507794958 | up |
| ENSG00000180422 | LINC00304 | 3.827966113 | up |
| ENSG00000227543 | SPAG5-AS1 | 3.827966113 | up |
| ENSG00000284879 | ENSG00000284879 | 3.827966113 | up |
| ENSG00000186193 | SAPCD2 | 1.278740222 | up |
| ENSG00000132002 | DNAJB1 | 1.277223436 | up |
| ENSG00000254634 | SMG1P6 | 3.173722546 | up |
| ENSG00000232656 | IDI2-AS1 | 2.21024128 | up |
| ENSG00000119737 | GPR75 | 2.21024128 | up |
| ENSG00000260260 | SNHG19 | 1.687369026 | up |
| ENSG00000226318 | RPS3AP38 | 6.517890568 | up |
| ENSG00000169246 | NPIPB3 | 6.517890568 | up |
| ENSG00000115155 | OTOF | 6.517890568 | up |
| ENSG00000005700 | IBTK | 1.362218056 | up |
| ENSG00000152894 | PTPRK | 1.521156467 | up |
| ENSG00000140451 | PIF1 | 1.31034139 | up |
| ENSG00000143801 | PSEN2 | 2.110710942 | up |
| ENSG00000090539 | CHRD | 2.110710942 | up |
| ENSG00000243716 | NPIPB5 | 1.413618194 | up |
| ENSG00000235217 | TSPY26P | 2.376301347 | up |
| ENSG00000182310 | SPACA6 | 1.934128117 | up |
| ENSG00000121653 | MAPK8IP1 | 3.729257255 | up |
| ENSG00000171931 | FBXW10 | 3.729257255 | up |
| ENSG00000203799 | CCDC162P | 3.729257255 | up |
| ENSG00000103423 | DNAJA3 | 1.31460838 | up |
| ENSG00000163491 | NEK10 | 1.610912049 | up |
| ENSG00000131848 | ZSCAN5A | 1.31245002 | up |
| ENSG00000163395 | IGFN1 | 2.114413907 | up |
| ENSG00000215704 | CELA2B | 6.381960509 | up |
| ENSG00000250067 | YJEFN3 | 6.381960509 | up |
| ENSG00000105737 | GRIK5 | 6.381960509 | up |
| ENSG00000288622 | PDCD6-AHRR | 6.381960509 | up |
| ENSG00000120694 | HSPH1 | 1.190893127 | up |
| ENSG00000259856 | RAB43P1 | 2.438999229 | up |
| ENSG00000210174 | MT-TR | 1.432948398 | up |
| ENSG00000117399 | CDC20 | 1.18521344 | up |
| ENSG00000175564 | UCP3 | 2.17562611 | up |
| ENSG00000101104 | PABPC1L | 2.17562611 | up |
| ENSG00000214783 | POLR2J4 | 1.799720097 | up |
| ENSG00000168389 | MFSD2A | 1.633049831 | up |
| ENSG00000109511 | ANXA10 | 1.943324987 | up |
| ENSG00000136161 | RCBTB2 | 1.469897847 | up |
| ENSG00000274372 | LINC02804 | 3.005004773 | up |
| ENSG00000100038 | TOP3B | 3.005004773 | up |
| ENSG00000243244 | STON1 | 3.62329548 | up |
| ENSG00000123977 | DAW1 | 3.62329548 | up |
| ENSG00000108691 | CCL2 | 1.630791266 | up |
| ENSG00000154040 | CABYR | 1.630791266 | up |
| ENSG00000173391 | OLR1 | 1.163469758 | up |
| ENSG00000198838 | RYR3 | 1.518865288 | up |
| ENSG00000138778 | CENPE | 1.159277639 | up |
| ENSG00000241322 | FBXW10B | 1.973735942 | up |
| ENSG00000173918 | C1QTNF1 | 1.241984691 | up |
| ENSG00000288917 | ENSG00000288917 | 2.369024318 | up |
| ENSG00000254093 | PINX1 | 2.369024318 | up |
| ENSG00000266680 | ENSG00000266680 | 1.420256778 | up |
| ENSG00000196943 | NOP9 | 1.285910441 | up |
| ENSG00000130558 | OLFM1 | 1.134413792 | up |
| ENSG00000124216 | SNAI1 | 1.226758363 | up |
| ENSG00000123975 | CKS2 | 1.138305188 | up |
| ENSG00000213918 | DNASE1 | 1.273710065 | up |
| ENSG00000254206 | NPIPB11 | 2.011765701 | up |
| ENSG00000196371 | FUT4 | 2.618118754 | up |
| ENSG00000139155 | SLCO1C1 | 2.618118754 | up |
| ENSG00000161647 | MPP3 | 1.148795073 | up |
| ENSG00000213199 | ASIC3 | 1.281322204 | up |
| ENSG00000055732 | MCOLN3 | 6.231878212 | up |
| ENSG00000215086 | NPM1P24 | 6.231878212 | up |
| ENSG00000227492 | CHUK-DT | 6.231878212 | up |
| ENSG00000284922 | LRTOMT | 6.231878212 | up |
| ENSG00000139174 | PRICKLE1 | 6.231878212 | up |
| ENSG00000160117 | ANKLE1 | 6.231878212 | up |
| ENSG00000284431 | ENSG00000284431 | 6.231878212 | up |
| ENSG00000131379 | C3orf20 | 6.231878212 | up |
| ENSG00000230533 | LINC03004 | 6.231878212 | up |
| ENSG00000085514 | PILRA | 6.231878212 | up |
| ENSG00000134057 | CCNB1 | 1.115008219 | up |
| ENSG00000291110 | TRIM16L | 1.141619071 | up |
| ENSG00000143554 | SLC27A3 | 2.912619221 | up |
| ENSG00000261884 | ENSG00000261884 | 2.912619221 | up |
| ENSG00000095752 | IL11 | 1.205723977 | up |
| ENSG00000140961 | OSGIN1 | 1.23371611 | up |
| ENSG00000258608 | DNAJC19P9 | 3.508929801 | up |
| ENSG00000229951 | FOSL2-AS1 | 3.508929801 | up |
| ENSG00000241839 | PLEKHO2 | 1.299066509 | up |
| ENSG00000117228 | GBP1 | 1.697866677 | up |
| ENSG00000188199 | NUTM2B | 2.126089515 | up |
| ENSG00000175592 | FOSL1 | 1.14262219 | up |
| ENSG00000272153 | ENSG00000272153 | 2.536224827 | up |
| ENSG00000170801 | HTRA3 | 1.343325905 | up |
| ENSG00000261008 | LINC01572 | 1.999622361 | up |
| ENSG00000260193 | LINC02846 | 1.388384757 | up |
| ENSG00000075218 | GTSE1 | 1.060593674 | up |
| ENSG00000151929 | BAG3 | 1.054774106 | up |
| ENSG00000256940 | PPP1R14B-AS1 | 1.901250748 | up |
| ENSG00000286207 | ENSG00000286207 | 1.901250748 | up |
| ENSG00000178035 | IMPDH2 | 1.214455559 | up |
| ENSG00000161692 | DBF4B | 1.060381593 | up |
| ENSG00000159588 | CCDC17 | 2.813910363 | up |
| ENSG00000178440 | TIMM23B-AGAP6 | 2.813910363 | up |
| ENSG00000239332 | LINC01119 | 2.813910363 | up |
| ENSG00000035499 | DEPDC1B | 1.064198302 | up |
| ENSG00000160072 | ATAD3B | 1.046226936 | up |
| ENSG00000275882 | IKBKGP1 | 1.303086365 | up |
| ENSG00000224420 | ADM5 | 1.822378558 | up |
| ENSG00000217644 | ENSG00000217644 | 6.064350616 | up |
| ENSG00000050628 | PTGER3 | 6.064350616 | up |
| ENSG00000229739 | PDC-AS1 | 6.064350616 | up |
| ENSG00000257225 | ENSG00000257225 | 6.064350616 | up |
| ENSG00000290394 | ENSG00000290394 | 6.064350616 | up |
| ENSG00000171903 | CYP4F11 | 6.064350616 | up |
| ENSG00000248663 | LINC00992 | 6.064350616 | up |
| ENSG00000274997 | H2AC12 | 6.064350616 | up |
| ENSG00000198576 | ARC | 6.064350616 | up |
| ENSG00000290923 | PGM5P2 | 6.064350616 | up |
| ENSG00000113161 | HMGCR | 1.035470844 | up |
| ENSG00000224635 | ENSG00000224635 | 1.75764081 | up |
| ENSG00000188766 | SPRED3 | 2.056114605 | up |
| ENSG00000150637 | CD226 | 3.384711452 | up |
| ENSG00000197646 | PDCD1LG2 | 3.384711452 | up |
| ENSG00000271833 | ENSG00000271833 | 3.384711452 | up |
| ENSG00000047648 | ARHGAP6 | 3.384711452 | up |
| ENSG00000165494 | PCF11 | 1.03738421 | up |
| ENSG00000081985 | IL12RB2 | 1.703499549 | up |
| ENSG00000273230 | ENSG00000273230 | 1.273574036 | up |
| ENSG00000288612 | ENSG00000288612 | 1.386709689 | up |
| ENSG00000128422 | KRT17 | 1.017521907 | up |
| ENSG00000188229 | TUBB4B | 1.017870792 | up |
| ENSG00000215908 | CROCCP2 | 1.935836186 | up |
| ENSG00000184492 | FOXD4L1 | 1.935836186 | up |
| ENSG00000157227 | MMP14 | 2.449400981 | up |
| ENSG00000238273 | ENSG00000238273 | 2.449400981 | up |
| ENSG00000125046 | SSUH2 | 2.449400981 | up |
| ENSG00000261924 | ENSG00000261924 | 1.454709109 | up |
| ENSG00000198298 | ZNF485 | 1.270467843 | up |
| ENSG00000250731 | TPM3P6 | 1.842647431 | up |
| ENSG00000287865 | ENSG00000287865 | 1.842647431 | up |
| ENSG00000163535 | SGO2 | 1.035628902 | up |
| ENSG00000203814 | H2BC18 | 1.434298069 | up |
| ENSG00000157653 | C9orf43 | 1.434298069 | up |
| ENSG00000059145 | UNKL | 1.040749991 | up |
| ENSG00000147383 | NSDHL | 1.018360411 | up |
| ENSG00000179776 | CDH5 | 1.166042912 | up |
| ENSG00000286431 | ENSG00000286431 | 1.399574729 | up |
| ENSG00000179304 | FAM156B | 1.768179048 | up |
| ENSG00000138172 | CALHM2 | 1.358761178 | up |
| ENSG00000171786 | NHLH1 | 2.217987735 | up |
| ENSG00000156510 | HKDC1 | 2.217987735 | up |
| ENSG00000255774 | LINC02747 | 2.217987735 | up |
| ENSG00000111665 | CDCA3 | 1.009181764 | up |
| ENSG00000073849 | ST6GAL1 | 1.29377073 | up |
| ENSG00000023902 | PLEKHO1 | 1.241353263 | up |
| ENSG00000196139 | AKR1C3 | 1.222180982 | up |
| ENSG00000286064 | ENSG00000286064 | 1.187568067 | up |
| ENSG00000180815 | MAP3K15 | 2.707948588 | up |
| ENSG00000244490 | RWDD4P1 | 1.544169633 | up |
| ENSG00000175768 | TOMM5 | 1.544169633 | up |
| ENSG00000269335 | IKBKG | 1.048371989 | up |
| ENSG00000177076 | ACER2 | 1.116910923 | up |
| ENSG00000263235 | ENSG00000263235 | 1.417352174 | up |
| ENSG00000224043 | CCNT2-AS1 | 1.516011878 | up |
| ENSG00000238197 | PAXBP1-AS1 | 1.3987806 | up |
| ENSG00000261342 | ENSG00000261342 | 1.38217629 | up |
| ENSG00000111348 | ARHGDIB | 1.218517619 | up |
| ENSG00000171522 | PTGER4 | 1.244727341 | up |
| ENSG00000169330 | MINAR1 | 1.262082283 | up |
| ENSG00000162006 | MSLNL | 1.869098836 | up |
| ENSG00000227440 | ATP5MC1P4 | 1.869098836 | up |
| ENSG00000176387 | HSD11B2 | 1.121032293 | up |
| ENSG00000137267 | TUBB2A | 1.003990918 | up |
| ENSG00000165995 | CACNB2 | 1.030815574 | up |
| ENSG00000288538 | ENSG00000288538 | 2.357015429 | up |
| ENSG00000236540 | ENSG00000236540 | 2.357015429 | up |
| ENSG00000271737 | ENSG00000271737 | 2.357015429 | up |
| ENSG00000102053 | ZC3H12B | 1.607672176 | up |
| ENSG00000213398 | LCAT | 1.227084471 | up |
| ENSG00000162482 | AKR7A3 | 3.248781393 | up |
| ENSG00000227741 | ENSG00000227741 | 3.248781393 | up |
| ENSG00000259424 | IRAIN | 3.248781393 | up |
| ENSG00000183604 | SMG1P5 | 3.248781393 | up |
| ENSG00000134775 | FHOD3 | 3.248781393 | up |
| ENSG00000278611 | ZNF426-DT | 3.248781393 | up |
| ENSG00000288958 | ENSG00000288958 | 3.248781393 | up |
| ENSG00000250321 | ENSG00000250321 | 3.248781393 | up |
| ENSG00000188993 | LRRC66 | 3.248781393 | up |
| ENSG00000284052 | ENSG00000284052 | 1.567662719 | up |
| ENSG00000159231 | CBR3 | 1.292046297 | up |
| ENSG00000272438 | ENSG00000272438 | 5.874782073 | up |
| ENSG00000284773 | ENSG00000284773 | 5.874782073 | up |
| ENSG00000235748 | SEPTIN14P12 | 5.874782073 | up |
| ENSG00000258539 | ENSG00000258539 | 5.874782073 | up |
| ENSG00000260490 | MYL12BP1 | 5.874782073 | up |
| ENSG00000259438 | MAPK6-DT | 5.874782073 | up |
| ENSG00000182175 | RGMA | 5.874782073 | up |
| ENSG00000277957 | SENP3-EIF4A1 | 5.874782073 | up |
| ENSG00000265474 | ENSG00000265474 | 5.874782073 | up |
| ENSG00000204866 | IGFL2 | 5.874782073 | up |
| ENSG00000258881 | ENSG00000258881 | 5.874782073 | up |
| ENSG00000230506 | ENSG00000230506 | 5.874782073 | up |
| ENSG00000101417 | PXMP4 | 5.874782073 | up |
| ENSG00000226258 | GRM7-AS3 | 5.874782073 | up |
| ENSG00000145087 | STXBP5L | 5.874782073 | up |
| ENSG00000248468 | ENSG00000248468 | 5.874782073 | up |
| ENSG00000251009 | ENSG00000251009 | 5.874782073 | up |
| ENSG00000138759 | FRAS1 | 5.874782073 | up |
| ENSG00000223551 | TMSB4XP4 | 5.874782073 | up |
| ENSG00000142279 | WTIP | 1.126335168 | up |
| ENSG00000169203 | NPIPB12 | 1.982571925 | up |
| ENSG00000260912 | ENSG00000260912 | 1.150262581 | up |
| ENSG00000188234 | AGAP4 | 1.082188222 | up |
| ENSG00000133740 | E2F5 | 1.05902002 | up |
| ENSG00000144821 | MYH15 | 1.799123925 | up |
| ENSG00000241170 | RPL31P46 | 1.653260289 | up |
| ENSG00000159899 | NPR2 | 1.653260289 | up |
| ENSG00000206077 | ZDHHC11B | 1.339604799 | up |
| ENSG00000078081 | LAMP3 | 1.438264503 | up |
| ENSG00000151640 | DPYSL4 | 1.600793092 | up |
| ENSG00000139329 | LUM | 1.600793092 | up |
| ENSG00000239653 | PSMD6-AS2 | 1.600793092 | up |
| ENSG00000135441 | BLOC1S1 | 1.001499071 | up |
| ENSG00000274210 | RNVU1-27 | 2.593582909 | up |
| ENSG00000266014 | ENSG00000266014 | 2.593582909 | up |
| ENSG00000179111 | HES7 | 1.005896741 | up |
| ENSG00000210176 | MT-TH | 1.004466112 | up |
| ENSG00000146094 | DOK3 | 1.029421465 | up |
| ENSG00000164684 | ZNF704 | 1.148923136 | up |
| ENSG00000179698 | WDR97 | 2.258306571 | up |
| ENSG00000116977 | LGALS8 | 1.08181132 | up |
| ENSG00000174028 | FAM3C2P | 1.033543979 | up |
| ENSG00000149591 | TAGLN | 1.275580798 | up |
| ENSG00000110446 | SLC15A3 | 2.049269961 | up |
| ENSG00000161835 | TAMALIN | 2.049269961 | up |
| ENSG00000230701 | FBXW4P1 | 2.049269961 | up |
| ENSG00000206503 | HLA-A | 2.049269961 | up |
| ENSG00000259865 | ENSG00000259865 | 1.419079335 | up |
| ENSG00000260708 | TBC1D22A-DT | 1.195162991 | up |
| ENSG00000250519 | ENSG00000250519 | 1.443522222 | up |
| ENSG00000137880 | GCHFR | 1.471835346 | up |
| ENSG00000101162 | TUBB1 | 1.905078022 | up |
| ENSG00000245025 | ENSG00000245025 | 1.905078022 | up |
| ENSG00000183598 | H3C13 | 1.505023969 | up |
| ENSG00000131471 | AOC3 | 1.10072556 | up |
| ENSG00000140403 | DNAJA4 | 1.592175299 | up |
| ENSG00000127366 | TAS2R5 | 1.592175299 | up |
| ENSG00000148204 | CRB2 | 1.592175299 | up |
| ENSG00000163141 | BNIPL | 1.651038916 | up |
| ENSG00000158156 | XKR8 | 3.098699096 | up |
| ENSG00000033122 | LRRC7 | 3.098699096 | up |
| ENSG00000259345 | ENSG00000259345 | 3.098699096 | up |
| ENSG00000261056 | ENSG00000261056 | 3.098699096 | up |
| ENSG00000277310 | ENSG00000277310 | 3.098699096 | up |
| ENSG00000147145 | LPAR4 | 3.098699096 | up |
| ENSG00000291156 | ENSG00000291156 | 1.10218353 | up |
| ENSG00000182359 | KBTBD3 | 1.16721448 | up |
| ENSG00000232973 | CYP1B1-AS1 | 1.16721448 | up |
| ENSG00000100060 | MFNG | 1.247262305 | up |
| ENSG00000113070 | HBEGF | 1.034143334 | up |
| ENSG00000250490 | LINC02145 | 1.3748504 | up |
| ENSG00000230424 | EMC1-AS1 | 5.65648152 | up |
| ENSG00000229447 | ENSG00000229447 | 5.65648152 | up |
| ENSG00000224805 | LINC00853 | 5.65648152 | up |
| ENSG00000201699 | RNVU1-24 | 5.65648152 | up |
| ENSG00000231073 | ENSG00000231073 | 5.65648152 | up |
| ENSG00000228169 | PPIAP19 | 5.65648152 | up |
| ENSG00000236991 | EDRF1-AS1 | 5.65648152 | up |
| ENSG00000265395 | MIR3944 | 5.65648152 | up |
| ENSG00000255669 | ENSG00000255669 | 5.65648152 | up |
| ENSG00000238121 | LINC00426 | 5.65648152 | up |
| ENSG00000272533 | SNORA28 | 5.65648152 | up |
| ENSG00000243904 | RPSAP5 | 5.65648152 | up |
| ENSG00000276107 | THBS1-IT1 | 5.65648152 | up |
| ENSG00000276571 | ENSG00000276571 | 5.65648152 | up |
| ENSG00000291066 | ENSG00000291066 | 5.65648152 | up |
| ENSG00000166592 | RRAD | 5.65648152 | up |
| ENSG00000291051 | ENSG00000291051 | 5.65648152 | up |
| ENSG00000227495 | KIF1C-AS1 | 5.65648152 | up |
| ENSG00000266126 | ENSG00000266126 | 5.65648152 | up |
| ENSG00000280212 | ENSG00000280212 | 5.65648152 | up |
| ENSG00000266844 | ENSG00000266844 | 5.65648152 | up |
| ENSG00000287766 | ENSG00000287766 | 5.65648152 | up |
| ENSG00000226763 | SRRM5 | 5.65648152 | up |
| ENSG00000197380 | DACT3 | 5.65648152 | up |
| ENSG00000268307 | LINC02560 | 5.65648152 | up |
| ENSG00000224559 | LINC01087 | 5.65648152 | up |
| ENSG00000196074 | SYCP2 | 5.65648152 | up |
| ENSG00000182586 | LINC00334 | 5.65648152 | up |
| ENSG00000227201 | CNN2P1 | 5.65648152 | up |
| ENSG00000128310 | GALR3 | 5.65648152 | up |
| ENSG00000231443 | ENSG00000231443 | 5.65648152 | up |
| ENSG00000183423 | LRIT3 | 5.65648152 | up |
| ENSG00000270681 | ENSG00000270681 | 5.65648152 | up |
| ENSG00000168412 | MTNR1A | 5.65648152 | up |
| ENSG00000250492 | INTS6P1 | 5.65648152 | up |
| ENSG00000213830 | CFL1P5 | 5.65648152 | up |
| ENSG00000249791 | ENSG00000249791 | 5.65648152 | up |
| ENSG00000248445 | SEMA6A-AS1 | 5.65648152 | up |
| ENSG00000273712 | ENSG00000273712 | 5.65648152 | up |
| ENSG00000091879 | ANGPT2 | 5.65648152 | up |
| ENSG00000257524 | ST6GALNAC6 | 5.65648152 | up |
| ENSG00000213212 | NCLP1 | 5.65648152 | up |
| ENSG00000165694 | FRMD7 | 5.65648152 | up |
| ENSG00000213397 | HAUS7 | 5.65648152 | up |
| ENSG00000109063 | MYH3 | 1.421423982 | up |
| ENSG00000265800 | ENSG00000265800 | 2.46936456 | up |
| ENSG00000289296 | ENSG00000289296 | 2.46936456 | up |
| ENSG00000290474 | GUSBP2 | 2.46936456 | up |
| ENSG00000238098 | ABCA17P | 1.450824458 | up |
| ENSG00000102385 | DRP2 | 1.193720704 | up |
| ENSG00000196873 | CBWD3 | 1.059823874 | up |
| ENSG00000234684 | SDCBP2-AS1 | 1.199873841 | up |
| ENSG00000273204 | ENSG00000273204 | 2.152344796 | up |
| ENSG00000110076 | NRXN2 | 2.152344796 | up |
| ENSG00000137804 | NUSAP1 | 2.152344796 | up |
| ENSG00000270091 | ENSG00000270091 | 2.152344796 | up |
| ENSG00000117242 | PINK1-AS | 1.080545028 | up |
| ENSG00000274290 | H2BC6 | 1.581064006 | up |
| ENSG00000270110 | ENSG00000270110 | 1.725581246 | up |
| ENSG00000092421 | SEMA6A | 1.725581246 | up |
| ENSG00000146221 | TCTE1 | 1.725581246 | up |
| ENSG00000157999 | ANKRD61 | 1.725581246 | up |
| ENSG00000285923 | ENSG00000285923 | 1.956884409 | up |
| ENSG00000254990 | ENSG00000254990 | 1.956884409 | up |
| ENSG00000118557 | PMFBP1 | 1.956884409 | up |
| ENSG00000244161 | FLNB-AS1 | 1.956884409 | up |
| ENSG00000145147 | SLIT2 | 1.956884409 | up |
| ENSG00000280047 | ENSG00000280047 | 1.956884409 | up |
| ENSG00000288999 | ENSG00000288999 | 1.823184095 | up |
| ENSG00000229927 | RHEBP1 | 1.823184095 | up |
| ENSG00000095539 | SEMA4G | 1.823184095 | up |
| ENSG00000175505 | CLCF1 | 1.04074588 | up |
| ENSG00000157870 | PRXL2B | 1.313174205 | up |
| ENSG00000087494 | PTHLH | 1.313174205 | up |
| ENSG00000215270 | TOMM40P2 | 1.313174205 | up |
| ENSG00000273568 | ENSG00000273568 | 1.329222531 | up |
| ENSG00000173531 | MST1 | 1.109638768 | up |
| ENSG00000258725 | PRC1-AS1 | 1.347694848 | up |

| Uniprot accession No. | Gene | Log_2_FoldChange | Down |
| --- | --- | --- | --- |
| ENSG00000128965 | CHAC1 | -3.269231098 | down |
| ENSG00000139269 | INHBE | -3.598168022 | down |
| ENSG00000130487 | KLHDC7B | -4.524157581 | down |
| ENSG00000128165 | ADM2 | -2.767337503 | down |
| ENSG00000237649 | KIFC1 | -3.086195258 | down |
| ENSG00000092010 | PSME1 | -2.489953621 | down |
| ENSG00000105550 | FGF21 | -8.093170165 | down |
| ENSG00000166046 | TCP11L2 | -3.103989849 | down |
| ENSG00000113739 | STC2 | -2.190855004 | down |
| ENSG00000112514 | CUTA | -2.271150391 | down |
| ENSG00000159314 | ARHGAP27 | -3.65757461 | down |
| ENSG00000111981 | ULBP1 | -2.285955185 | down |
| ENSG00000175643 | RMI2 | -2.182880102 | down |
| ENSG00000160179 | ABCG1 | -2.200979386 | down |
| ENSG00000054282 | SDCCAG8 | -7.72033916 | down |
| ENSG00000012963 | UBR7 | -2.384028463 | down |
| ENSG00000174951 | FUT1 | -2.289071418 | down |
| ENSG00000100889 | PCK2 | -2.44148795 | down |
| ENSG00000271992 | ENSG00000271992 | -2.944989531 | down |
| ENSG00000110768 | GTF2H1 | -2.045631084 | down |
| ENSG00000147689 | FAM83A | -1.938871296 | down |
| ENSG00000137393 | RNF144B | -1.989925005 | down |
| ENSG00000116852 | KIF21B | -1.902644894 | down |
| ENSG00000261770 | ENSG00000261770 | -2.842132515 | down |
| ENSG00000170291 | ELP5 | -2.061110766 | down |
| ENSG00000102984 | ZNF821 | -2.730386018 | down |
| ENSG00000119632 | IFI27L2 | -3.448861214 | down |
| ENSG00000070669 | ASNS | -2.350138696 | down |
| ENSG00000187801 | ZFP69B | -1.960379231 | down |
| ENSG00000176046 | NUPR1 | -2.007142127 | down |
| ENSG00000139197 | PEX5 | -1.827455488 | down |
| ENSG00000146733 | PSPH | -1.687865655 | down |
| ENSG00000277449 | CEBPB-AS1 | -3.04791554 | down |
| ENSG00000118412 | CASP8AP2 | -2.458134018 | down |
| ENSG00000259230 | LINC02323 | -2.010807022 | down |
| ENSG00000265972 | TXNIP | -1.710871378 | down |
| ENSG00000189060 | H1-0 | -1.659880253 | down |
| ENSG00000157514 | TSC22D3 | -1.671361266 | down |
| ENSG00000170270 | GON7 | -2.483067487 | down |
| ENSG00000101255 | TRIB3 | -1.604372501 | down |
| ENSG00000116761 | CTH | -1.617587017 | down |
| ENSG00000280649 | ENSG00000280649 | -4.151842994 | down |
| ENSG00000164463 | CREBRF | -1.86146047 | down |
| ENSG00000168209 | DDIT4 | -1.573371364 | down |
| ENSG00000274276 | ENSG00000274276 | -1.899626631 | down |
| ENSG00000149150 | SLC43A1 | -1.588313438 | down |
| ENSG00000243479 | MNX1-AS1 | -2.734509087 | down |
| ENSG00000105516 | DBP | -1.599731802 | down |
| ENSG00000171798 | KNDC1 | -2.480541714 | down |
| ENSG00000265666 | RARA-AS1 | -4.081892784 | down |
| ENSG00000182378 | PLCXD1 | -1.550455955 | down |
| ENSG00000282988 | ENSG00000282988 | -2.018665403 | down |
| ENSG00000133393 | CEP20 | -2.097428202 | down |
| ENSG00000260920 | ENSG00000260920 | -1.707491119 | down |
| ENSG00000107731 | UNC5B | -1.598003833 | down |
| ENSG00000105327 | BBC3 | -1.706943328 | down |
| ENSG00000228626 | ENSG00000228626 | -6.877804715 | down |
| ENSG00000262468 | LINC01569 | -2.445883738 | down |
| ENSG00000261824 | LINC00662 | -1.549949564 | down |
| ENSG00000215262 | KCNU1 | -4.008377388 | down |
| ENSG00000166123 | GPT2 | -1.496155063 | down |
| ENSG00000240875 | LINC00886 | -1.910135997 | down |
| ENSG00000231527 | FAM27C | -2.324818052 | down |
| ENSG00000142459 | EVI5L | -1.555573609 | down |
| ENSG00000288930 | ENSG00000288930 | -2.165457594 | down |
| ENSG00000164284 | GRPEL2 | -1.480593253 | down |
| ENSG00000227630 | LINC01132 | -6.779145011 | down |
| ENSG00000170464 | DNAJC18 | -6.779145011 | down |
| ENSG00000188095 | MESP2 | -3.93091378 | down |
| ENSG00000290877 | ROCK1P1 | -3.93091378 | down |
| ENSG00000224177 | LINC00570 | -1.995253077 | down |
| ENSG00000184451 | CCR10 | -2.737210932 | down |
| ENSG00000169169 | CPT1C | -2.56522605 | down |
| ENSG00000290854 | ENSG00000290854 | -2.436688645 | down |
| ENSG00000281005 | LINC00921 | -2.436688645 | down |
| ENSG00000198835 | GJC2 | -1.890142981 | down |
| ENSG00000273270 | ENSG00000273270 | -2.104224695 | down |
| ENSG00000247735 | KCTD13-DT | -2.686812388 | down |
| ENSG00000134830 | C5AR2 | -1.977671892 | down |
| ENSG00000153982 | GDPD1 | -1.504870626 | down |
| ENSG00000114796 | KLHL24 | -1.443249578 | down |
| ENSG00000255135 | EMSY-DT | -1.742939666 | down |
| ENSG00000162733 | DDR2 | -1.667108969 | down |
| ENSG00000272405 | ENSG00000272405 | -1.424576276 | down |
| ENSG00000154359 | LONRF1 | -2.258883316 | down |
| ENSG00000227764 | LINC01693 | -2.866464857 | down |
| ENSG00000260267 | ENSG00000260267 | -1.566992926 | down |
| ENSG00000100311 | PDGFB | -1.598163979 | down |
| ENSG00000181135 | ZNF707 | -1.797268358 | down |
| ENSG00000253276 | CCDC71L | -1.710197597 | down |
| ENSG00000178093 | TSSK6 | -1.718250913 | down |
| ENSG00000181885 | CLDN7 | -3.762267869 | down |
| ENSG00000270412 | ENSG00000270412 | -3.762267869 | down |
| ENSG00000106823 | ECM2 | -1.726950184 | down |
| ENSG00000257906 | LINC02156 | -6.55894019 | down |
| ENSG00000272323 | TTC23L-AS1 | -6.55894019 | down |
| ENSG00000232818 | RPS2P32 | -1.777673846 | down |
| ENSG00000143365 | RORC | -1.444402393 | down |
| ENSG00000107864 | CPEB3 | -1.444612327 | down |
| ENSG00000116285 | ERRFI1 | -1.371843871 | down |
| ENSG00000081181 | ARG2 | -1.457922672 | down |
| ENSG00000165879 | FRAT1 | -1.530095184 | down |
| ENSG00000130513 | GDF15 | -1.368493825 | down |
| ENSG00000205143 | ARID3C | -3.088637733 | down |
| ENSG00000229953 | ENSG00000229953 | -1.616179769 | down |
| ENSG00000108306 | FBXL20 | -1.346011064 | down |
| ENSG00000230513 | THAP7-AS1 | -1.737667893 | down |
| ENSG00000141738 | GRB7 | -1.498416182 | down |
| ENSG00000165171 | METTL27 | -1.608521165 | down |
| ENSG00000205808 | PLPP6 | -1.509630316 | down |
| ENSG00000285644 | ENSG00000285644 | -3.669925374 | down |
| ENSG00000235560 | ZNF747-DT | -3.669925374 | down |
| ENSG00000065361 | ERBB3 | -1.538293236 | down |
| ENSG00000244731 | C4A | -2.524105176 | down |
| ENSG00000115902 | SLC1A4 | -1.292572884 | down |
| ENSG00000005961 | ITGA2B | -3.011174125 | down |
| ENSG00000176597 | B3GNT5 | -3.011174125 | down |
| ENSG00000289852 | ENSG00000289852 | -1.844076287 | down |
| ENSG00000259330 | INAFM2 | -1.399839506 | down |
| ENSG00000110619 | CARS1 | -1.313054653 | down |
| ENSG00000172216 | CEBPB | -1.26870196 | down |
| ENSG00000126368 | NR1D1 | -1.300105035 | down |
| ENSG00000290926 | LINC03006 | -1.979519303 | down |
| ENSG00000135069 | PSAT1 | -1.258299558 | down |
| ENSG00000175938 | ORAI3 | -1.8712781 | down |
| ENSG00000181852 | RNF41 | -1.271111686 | down |
| ENSG00000177706 | FAM20C | -1.706055225 | down |
| ENSG00000179862 | CITED4 | -1.26319119 | down |
| ENSG00000273812 | LINC02970 | -1.78217923 | down |
| ENSG00000109536 | FRG1 | -2.321182858 | down |
| ENSG00000135116 | HRK | -1.903194197 | down |
| ENSG00000187017 | ESPN | -1.81245683 | down |
| ENSG00000153879 | CEBPG | -1.254080219 | down |
| ENSG00000253616 | ENSG00000253616 | -1.83743322 | down |
| ENSG00000251192 | ZNF674 | -1.401289453 | down |
| ENSG00000132470 | ITGB4 | -1.549132643 | down |
| ENSG00000249996 | PPIC-AS1 | -3.571265671 | down |
| ENSG00000289511 | ENSG00000289511 | -2.604967949 | down |
| ENSG00000154118 | JPH3 | -1.755769359 | down |
| ENSG00000185105 | MYADML2 | -2.929314031 | down |
| ENSG00000106105 | GARS1 | -1.213415698 | down |
| ENSG00000189334 | S100A14 | -1.469989577 | down |
| ENSG00000187630 | DHRS4L2 | -1.401267216 | down |
| ENSG00000181350 | LRRC75A | -1.552902073 | down |
| ENSG00000250208 | FZD10-AS1 | -1.380573556 | down |
| ENSG00000167074 | TEF | -1.260073855 | down |
| ENSG00000175197 | DDIT3 | -1.313911467 | down |
| ENSG00000237036 | ZEB1-AS1 | -1.455932035 | down |
| ENSG00000267216 | ZNF8-ERVK3-1 | -1.802775243 | down |
| ENSG00000285793 | ANAPC1P2 | -1.802775243 | down |
| ENSG00000229956 | ZRANB2-DT | -6.298962863 | down |
| ENSG00000289088 | ENSG00000289088 | -6.298962863 | down |
| ENSG00000249286 | AMD1P3 | -6.298962863 | down |
| ENSG00000164741 | DLC1 | -6.298962863 | down |
| ENSG00000188959 | C9orf152 | -6.298962863 | down |
| ENSG00000175906 | ARL4D | -1.242714196 | down |
| ENSG00000075651 | PLD1 | -1.255396801 | down |
| ENSG00000227467 | LINC01537 | -1.340575398 | down |
| ENSG00000130517 | PGPEP1 | -1.213471005 | down |
| ENSG00000188263 | IL17REL | -1.698091151 | down |
| ENSG00000272667 | ENSG00000272667 | -2.531452553 | down |
| ENSG00000239388 | ASB14 | -2.531452553 | down |
| ENSG00000065060 | UHRF1BP1 | -1.17079818 | down |
| ENSG00000276855 | ENSG00000276855 | -1.448325262 | down |
| ENSG00000258708 | SLC25A21-AS1 | -2.842528214 | down |
| ENSG00000121104 | FAM117A | -3.465360541 | down |
| ENSG00000150636 | CCDC102B | -3.465360541 | down |
| ENSG00000244968 | LIFR-AS1 | -2.210698723 | down |
| ENSG00000289483 | ENSG00000289483 | -1.996875916 | down |
| ENSG00000185186 | LINC00313 | -1.996875916 | down |
| ENSG00000112812 | PRSS16 | -1.342770376 | down |
| ENSG00000198690 | FAN1 | -1.478001803 | down |
| ENSG00000023228 | NDUFS1 | -1.185941089 | down |
| ENSG00000167964 | RAB26 | -1.242641947 | down |
| ENSG00000174749 | FAM241A | -1.209787574 | down |
| ENSG00000236859 | NIFK-AS1 | -1.565531984 | down |
| ENSG00000143476 | DTL | -1.149009715 | down |
| ENSG00000100302 | RASD2 | -1.300420281 | down |
| ENSG00000271780 | ENSG00000271780 | -1.684981732 | down |
| ENSG00000108984 | MAP2K6 | -1.162131816 | down |
| ENSG00000123405 | NFE2 | -1.819627153 | down |
| ENSG00000155792 | DEPTOR | -1.252034288 | down |
| ENSG00000164967 | RPP25L | -1.216995708 | down |
| ENSG00000268573 | ENSG00000268573 | -1.240324893 | down |
| ENSG00000268575 | ENSG00000268575 | -1.632694072 | down |
| ENSG00000140044 | JDP2 | -1.148783041 | down |
| ENSG00000165985 | C1QL3 | -2.453988945 | down |
| ENSG00000007314 | SCN4A | -2.453988945 | down |
| ENSG00000121005 | CRISPLD1 | -2.453988945 | down |
| ENSG00000198056 | PRIM1 | -1.181346481 | down |
| ENSG00000246273 | SBF2-AS1 | -1.247290035 | down |
| ENSG00000008323 | PLEKHG6 | -2.15211273 | down |
| ENSG00000170298 | LGALS9B | -2.15211273 | down |
| ENSG00000117245 | KIF17 | -6.148994229 | down |
| ENSG00000276850 | ENSG00000276850 | -6.148994229 | down |
| ENSG00000275807 | ENSG00000275807 | -6.148994229 | down |
| ENSG00000230076 | RPL10P6 | -6.148994229 | down |
| ENSG00000172296 | SPTLC3 | -6.148994229 | down |
| ENSG00000289634 | ENSG00000289634 | -6.148994229 | down |
| ENSG00000280424 | ENSG00000280424 | -6.148994229 | down |
| ENSG00000183977 | PP2D1 | -6.148994229 | down |
| ENSG00000230454 | ENSG00000230454 | -6.148994229 | down |
| ENSG00000272744 | ENSG00000272744 | -6.148994229 | down |
| ENSG00000151882 | CCL28 | -6.148994229 | down |
| ENSG00000159904 | ZNF890P | -6.148994229 | down |
| ENSG00000243433 | ENSG00000243433 | -6.148994229 | down |
| ENSG00000172007 | RAB33B | -1.183711705 | down |
| ENSG00000170153 | RNF150 | -1.202571398 | down |
| ENSG00000009950 | MLXIPL | -1.22562626 | down |
| ENSG00000105784 | RUNDC3B | -2.750185719 | down |
| ENSG00000184208 | C22orf46 | -1.146706392 | down |
| ENSG00000162772 | ATF3 | -1.121168514 | down |
| ENSG00000087266 | SH3BP2 | -1.114647046 | down |
| ENSG00000141933 | TPGS1 | -1.272292564 | down |
| ENSG00000259319 | JDP2-AS1 | -2.009672948 | down |
| ENSG00000160200 | CBS | -1.125253874 | down |
| ENSG00000120915 | EPHX2 | -1.495557073 | down |
| ENSG00000128573 | FOXP2 | -1.404974925 | down |
| ENSG00000174567 | GOLT1A | -3.35106085 | down |
| ENSG00000179044 | EXOC3L1 | -3.35106085 | down |
| ENSG00000267073 | ENSG00000267073 | -3.35106085 | down |
| ENSG00000121577 | POPDC2 | -3.35106085 | down |
| ENSG00000227908 | IL6ST-DT | -3.35106085 | down |
| ENSG00000273841 | TAF9 | -3.35106085 | down |
| ENSG00000196972 | SMIM10L2B | -3.35106085 | down |
| ENSG00000272764 | ENSG00000272764 | -2.273972486 | down |
| ENSG00000254595 | ENSG00000254595 | -2.273972486 | down |
| ENSG00000257038 | ARHGEF17-AS1 | -2.273972486 | down |
| ENSG00000213213 | CCDC183 | -1.376205404 | down |
| ENSG00000087074 | PPP1R15A | -1.105433864 | down |
| ENSG00000264247 | ZNF407-AS1 | -1.263905557 | down |
| ENSG00000213189 | BTF3L4P2 | -1.358087375 | down |
| ENSG00000119801 | YPEL5 | -1.118026967 | down |
| ENSG00000287126 | ENSG00000287126 | -1.213230032 | down |
| ENSG00000186862 | PDZD7 | -1.43201411 | down |
| ENSG00000178607 | ERN1 | -1.144786323 | down |
| ENSG00000180066 | LINC02870 | -2.372128851 | down |
| ENSG00000290917 | RAET1K | -2.372128851 | down |
| ENSG00000132692 | BCAN | -1.397054321 | down |
| ENSG00000167733 | HSD11B1L | -1.494994177 | down |
| ENSG00000253320 | MAILR | -1.953373505 | down |
| ENSG00000254827 | SLC22A18AS | -1.592129377 | down |
| ENSG00000102981 | PARD6A | -1.271791415 | down |
| ENSG00000258701 | LINC00638 | -1.669544856 | down |
| ENSG00000103319 | EEF2K | -1.241709125 | down |
| ENSG00000197245 | FAM110D | -1.555981267 | down |
| ENSG00000263155 | MYZAP | -2.13050688 | down |
| ENSG00000291005 | NBPF25P | -2.651526016 | down |
| ENSG00000080031 | PTPRH | -1.467763676 | down |
| ENSG00000174804 | FZD4 | -1.110643444 | down |
| ENSG00000185567 | AHNAK2 | -1.052479784 | down |
| ENSG00000204237 | OXLD1 | -1.077325999 | down |
| ENSG00000171219 | CDC42BPG | -1.150587945 | down |
| ENSG00000168679 | SLC16A4 | -1.145033632 | down |
| ENSG00000289911 | ENSG00000289911 | -1.287777781 | down |
| ENSG00000234311 | ENSG00000234311 | -1.758610165 | down |
| ENSG00000162460 | TMEM82 | -5.981608273 | down |
| ENSG00000175318 | GRAMD2A | -5.981608273 | down |
| ENSG00000265095 | FTLP12 | -5.981608273 | down |
| ENSG00000273828 | ENSG00000273828 | -5.981608273 | down |
| ENSG00000227456 | LINC00310 | -5.981608273 | down |
| ENSG00000291096 | ENSG00000291096 | -5.981608273 | down |
| ENSG00000253327 | RAD21-AS1 | -5.981608273 | down |
| ENSG00000259891 | ENSG00000259891 | -1.686886773 | down |
| ENSG00000090861 | AARS1 | -1.027997431 | down |
| ENSG00000229021 | ENSG00000229021 | -3.226920352 | down |
| ENSG00000223509 | WHAMMP1 | -3.226920352 | down |
| ENSG00000279479 | ENSG00000279479 | -3.226920352 | down |
| ENSG00000104951 | IL4I1 | -3.226920352 | down |
| ENSG00000172478 | MAB21L4 | -3.226920352 | down |
| ENSG00000198865 | CCDC152 | -3.226920352 | down |
| ENSG00000176428 | VPS37D | -3.226920352 | down |
| ENSG00000290924 | ENSG00000290924 | -3.226920352 | down |
| ENSG00000233382 | NKAPP1 | -3.226920352 | down |
| ENSG00000168936 | TMEM129 | -1.047217885 | down |
| ENSG00000165030 | NFIL3 | -1.050600914 | down |
| ENSG00000228137 | ENSG00000228137 | -1.626640909 | down |
| ENSG00000180938 | ZNF572 | -1.440350115 | down |
| ENSG00000132846 | ZBED3 | -1.043985605 | down |
| ENSG00000176933 | TOB2P1 | -1.251017476 | down |
| ENSG00000135245 | HILPDA | -1.069800519 | down |
| ENSG00000105141 | CASP14 | -1.894787512 | down |
| ENSG00000289135 | ENSG00000289135 | -1.894787512 | down |
| ENSG00000251169 | LINC01843 | -1.894787512 | down |
| ENSG00000119139 | TJP2 | -1.401119496 | down |
| ENSG00000248092 | NNT-AS1 | -1.049614936 | down |
| ENSG00000203279 | ENSG00000203279 | -2.285343034 | down |
| ENSG00000170915 | PAQR8 | -1.382373522 | down |
| ENSG00000113369 | ARRDC3 | -1.033154201 | down |
| ENSG00000177606 | JUN | -1.02053428 | down |
| ENSG00000237248 | LINC00987 | -1.179430564 | down |
| ENSG00000092470 | WDR76 | -1.024025419 | down |
| ENSG00000168887 | C2orf68 | -1.024025467 | down |
| ENSG00000133317 | LGALS12 | -1.049318319 | down |
| ENSG00000135338 | LCA5 | -1.436732122 | down |
| ENSG00000290057 | ENSG00000290057 | -2.053043272 | down |
| ENSG00000247934 | ENSG00000247934 | -2.053043272 | down |
| ENSG00000232774 | LINC03033 | -2.053043272 | down |
| ENSG00000228812 | LAMA5-AS1 | -2.053043272 | down |
| ENSG00000187650 | VMAC | -1.639779776 | down |
| ENSG00000118513 | MYB | -1.286539369 | down |
| ENSG00000163995 | ABLIM2 | -1.163426924 | down |
| ENSG00000289005 | ENSG00000289005 | -2.545620886 | down |
| ENSG00000164451 | CALHM4 | -2.545620886 | down |
| ENSG00000215068 | ANXA2R-AS1 | -1.104749313 | down |
| ENSG00000250988 | SNHG21 | -1.960566033 | down |
| ENSG00000259291 | ZNF710-AS1 | -1.960566033 | down |
| ENSG00000114779 | ABHD14B | -1.0072936 | down |
| ENSG00000184939 | ZFP90 | -1.154782919 | down |
| ENSG00000272068 | BCAN-AS1 | -1.407063476 | down |
| ENSG00000221843 | C2orf16 | -1.254729356 | down |
| ENSG00000198945 | L3MBTL3 | -1.101613036 | down |
| ENSG00000100784 | RPS6KA5 | -1.002460169 | down |
| ENSG00000171160 | MORN4 | -1.735072952 | down |
| ENSG00000285184 | ENSG00000285184 | -1.655988544 | down |
| ENSG00000260552 | COSMOC | -1.163326527 | down |
| ENSG00000244176 | ENSG00000244176 | -2.193000539 | down |
| ENSG00000231841 | FAM192BP | -2.193000539 | down |
| ENSG00000213071 | LPAL2 | -2.193000539 | down |
| ENSG00000145687 | SSBP2 | -1.206499345 | down |
| ENSG00000104518 | GSDMD | -1.591082569 | down |
| ENSG00000152942 | RAD17 | -1.033519934 | down |
| ENSG00000251257 | ENSG00000251257 | -1.536804476 | down |
| ENSG00000149090 | PAMR1 | -3.091083522 | down |
| ENSG00000205861 | PCOTH | -3.091083522 | down |
| ENSG00000284308 | C2orf81 | -3.091083522 | down |
| ENSG00000183054 | RGPD6 | -3.091083522 | down |
| ENSG00000100385 | IL2RB | -3.091083522 | down |
| ENSG00000053524 | MCF2L2 | -3.091083522 | down |
| ENSG00000288741 | ENSG00000288741 | -3.091083522 | down |
| ENSG00000290114 | ENSG00000290114 | -3.091083522 | down |
| ENSG00000254027 | LNMICC | -3.091083522 | down |
| ENSG00000141294 | LRRC46 | -1.490710851 | down |
| ENSG00000236700 | LINC01010 | -1.490710851 | down |
| ENSG00000153029 | MR1 | -1.118272234 | down |
| ENSG00000267534 | S1PR2 | -1.118272234 | down |
| ENSG00000156500 | PABIR3 | -1.118272234 | down |
| ENSG00000138639 | ARHGAP24 | -1.325999504 | down |
| ENSG00000227775 | ENSG00000227775 | -1.451061248 | down |
| ENSG00000089505 | CMTM1 | -1.416580317 | down |
| ENSG00000250802 | ZBED3-AS1 | -1.416580317 | down |
| ENSG00000178226 | PRSS36 | -1.354838195 | down |
| ENSG00000162496 | DHRS3 | -1.049410765 | down |
| ENSG00000183250 | LINC01547 | -1.001664849 | down |
| ENSG00000224358 | TMCO1-AS1 | -5.792221131 | down |
| ENSG00000284634 | ENSG00000284634 | -5.792221131 | down |
| ENSG00000205693 | MANSC4 | -5.792221131 | down |
| ENSG00000257557 | PPP1R12A-AS1 | -5.792221131 | down |
| ENSG00000139656 | SMIM2 | -5.792221131 | down |
| ENSG00000279636 | LINC00216 | -5.792221131 | down |
| ENSG00000166145 | SPINT1 | -5.792221131 | down |
| ENSG00000270264 | NDUFB8P2 | -5.792221131 | down |
| ENSG00000267544 | HIKESHIP2 | -5.792221131 | down |
| ENSG00000179954 | SSC5D | -5.792221131 | down |
| ENSG00000233266 | HMGB1P31 | -5.792221131 | down |
| ENSG00000163219 | ARHGAP25 | -5.792221131 | down |
| ENSG00000232124 | ENSG00000232124 | -5.792221131 | down |
| ENSG00000251322 | SHANK3 | -5.792221131 | down |
| ENSG00000270021 | ENSG00000270021 | -5.792221131 | down |
| ENSG00000238035 | ENSG00000238035 | -5.792221131 | down |
| ENSG00000225945 | ZFAND3-DT | -5.792221131 | down |
| ENSG00000233231 | HNRNPA1P49 | -5.792221131 | down |
| ENSG00000136197 | C7orf25 | -5.792221131 | down |
| ENSG00000284948 | ENSG00000284948 | -5.792221131 | down |
| ENSG00000235609 | ENSG00000235609 | -1.971183177 | down |
| ENSG00000004838 | ZMYND10 | -1.971183177 | down |
| ENSG00000196569 | LAMA2 | -1.971183177 | down |
| ENSG00000271122 | HERPUD2-AS1 | -1.052260443 | down |
| ENSG00000165698 | SPACA9 | -1.125956928 | down |
| ENSG00000274180 | NATD1 | -1.096377311 | down |
| ENSG00000232859 | LYRM9 | -1.245345241 | down |
| ENSG00000290374 | FAM86EP | -1.099690855 | down |
| ENSG00000242715 | CCDC169 | -1.890615823 | down |
| ENSG00000289469 | ENSG00000289469 | -1.817100427 | down |
| ENSG00000223396 | RPS10P7 | -1.135709961 | down |
| ENSG00000205707 | ETFRF1 | -1.044538744 | down |
| ENSG00000262050 | ENSG00000262050 | -1.289313532 | down |
| ENSG00000271119 | ENSG00000271119 | -1.769955696 | down |
| ENSG00000130045 | NXNL2 | -1.769955696 | down |
| ENSG00000109220 | CHIC2 | -1.033055237 | down |
| ENSG00000122694 | GLIPR2 | -1.676486959 | down |
| ENSG00000163884 | KLF15 | -1.015586302 | down |
| ENSG00000240303 | ACAD11 | -1.394178559 | down |
| ENSG00000271335 | CCNY-AS1 | -1.175750458 | down |
| ENSG00000205559 | CHKB-DT | -1.348961145 | down |
| ENSG00000226137 | BAIAP2-DT | -1.034960882 | down |
| ENSG00000150773 | PIH1D2 | -1.377190793 | down |
| ENSG00000276223 | ENSG00000276223 | -1.540684024 | down |
| ENSG00000052344 | PRSS8 | -1.024418931 | down |
| ENSG00000281571 | ENSG00000281571 | -2.431321195 | down |
| ENSG00000166473 | PKD1L2 | -2.431321195 | down |
| ENSG00000200250 | RNU6-1147P | -2.431321195 | down |
| ENSG00000148357 | HMCN2 | -2.431321195 | down |
| ENSG00000170442 | KRT86 | -1.233706215 | down |
| ENSG00000284968 | ENSG00000284968 | -1.233706215 | down |
| ENSG00000127528 | KLF2 | -1.031773221 | down |
| ENSG00000154079 | SDHAF4 | -1.155608815 | down |
| ENSG00000166816 | LDHD | -1.163170955 | down |
| ENSG00000234614 | C2CD4D-AS1 | -2.094340835 | down |
| ENSG00000225518 | LINC01703 | -2.094340835 | down |
| ENSG00000101004 | NINL | -2.094340835 | down |
| ENSG00000276076 | ENSG00000276076 | -2.094340835 | down |
| ENSG00000177694 | NAALADL2 | -2.094340835 | down |
| ENSG00000119227 | PIGZ | -2.094340835 | down |
| ENSG00000271021 | ENSG00000271021 | -2.094340835 | down |
| ENSG00000167216 | KATNAL2 | -1.045957115 | down |
| ENSG00000092068 | SLC7A8 | -1.128632261 | down |
| ENSG00000289207 | ENSG00000289207 | -1.88439736 | down |
| ENSG00000236829 | ENSG00000236829 | -1.88439736 | down |
| ENSG00000237424 | FOXD2-AS1 | -1.135181986 | down |
| ENSG00000065923 | SLC9A7 | -1.024029302 | down |
| ENSG00000175175 | PPM1E | -1.400874417 | down |
| ENSG00000145431 | PDGFC | -1.400874417 | down |
| ENSG00000178199 | ZC3H12D | -1.400874417 | down |
| ENSG00000058673 | ZC3H11A | -1.739636819 | down |
| ENSG00000287935 | ENSG00000287935 | -1.739636819 | down |
| ENSG00000132141 | CCT6B | -1.739636819 | down |
| ENSG00000267424 | ENSG00000267424 | -1.739636819 | down |
| ENSG00000230154 | AIDAP1 | -1.739636819 | down |
| ENSG00000100290 | BIK | -1.739636819 | down |
| ENSG00000273145 | ENSG00000273145 | -1.441000272 | down |
| ENSG00000116183 | PAPPA2 | -1.488460947 | down |
| ENSG00000267100 | ILF3-DT | -1.029789046 | down |
| ENSG00000234362 | LINC01914 | -1.703240815 | down |
| ENSG00000197405 | C5AR1 | -1.092556671 | down |
| ENSG00000221944 | TIGD1 | -1.034312887 | down |
| ENSG00000273338 | ENSG00000273338 | -2.941114888 | down |
| ENSG00000245571 | FAM111A-DT | -2.941114888 | down |
| ENSG00000128872 | TMOD2 | -2.941114888 | down |
| ENSG00000259732 | ENSG00000259732 | -2.941114888 | down |
| ENSG00000260269 | ENSG00000260269 | -2.941114888 | down |
| ENSG00000259736 | CRTC3-AS1 | -2.941114888 | down |
| ENSG00000262703 | ENSG00000262703 | -2.941114888 | down |
| ENSG00000168589 | DYNLRB2 | -2.941114888 | down |
| ENSG00000273493 | ENSG00000273493 | -2.941114888 | down |
| ENSG00000251131 | ENSG00000251131 | -2.941114888 | down |
| ENSG00000279968 | CCDC28A-AS1 | -2.941114888 | down |
| ENSG00000184925 | LCN12 | -2.941114888 | down |
| ENSG00000267080 | ASB16-AS1 | -1.038497468 | down |
| ENSG00000221990 | EXOC3-AS1 | -1.038497468 | down |
| ENSG00000137310 | TCF19 | -1.301793007 | down |
| ENSG00000224281 | SLC25A5-AS1 | -1.301793007 | down |
| ENSG00000181894 | ZNF329 | -1.120815826 | down |
| ENSG00000214367 | HAUS3 | -1.06946794 | down |
| ENSG00000261455 | LINC01003 | -1.127590877 | down |
| ENSG00000160345 | PIERCE1 | -1.022051365 | down |
| ENSG00000147394 | ZNF185 | -1.134830289 | down |
| ENSG00000188747 | NOXA1 | -1.271229019 | down |
| ENSG00000100307 | CBX7 | -1.027356785 | down |
| ENSG00000172731 | LRRC20 | -1.026787386 | down |
| ENSG00000278970 | HEIH | -1.088063789 | down |
| ENSG00000196812 | ZSCAN16 | -1.013398758 | down |
| ENSG00000172346 | CSDC2 | -1.150907664 | down |
| ENSG00000174456 | C12orf76 | -1.0473129 | down |
| ENSG00000104081 | BMF | -1.159868072 | down |
| ENSG00000176371 | ZSCAN2 | -1.159868072 | down |
| ENSG00000116329 | OPRD1 | -5.574161211 | down |
| ENSG00000238287 | EXO5-DT | -5.574161211 | down |
| ENSG00000223393 | ENSG00000223393 | -5.574161211 | down |
| ENSG00000243742 | RPLP0P2 | -5.574161211 | down |
| ENSG00000278879 | ENSG00000278879 | -5.574161211 | down |
| ENSG00000227376 | FTH1P16 | -5.574161211 | down |
| ENSG00000248265 | FLJ12825 | -5.574161211 | down |
| ENSG00000277247 | ENSG00000277247 | -5.574161211 | down |
| ENSG00000241556 | ENSG00000241556 | -5.574161211 | down |
| ENSG00000289878 | ENSG00000289878 | -5.574161211 | down |
| ENSG00000224418 | STK24-AS1 | -5.574161211 | down |
| ENSG00000213937 | CLDN9 | -5.574161211 | down |
| ENSG00000103522 | IL21R | -5.574161211 | down |
| ENSG00000281856 | ENSG00000281856 | -5.574161211 | down |
| ENSG00000266830 | ENSG00000266830 | -5.574161211 | down |
| ENSG00000266456 | ENSG00000266456 | -5.574161211 | down |
| ENSG00000264859 | DSG2-AS1 | -5.574161211 | down |
| ENSG00000204334 | ERICH2 | -5.574161211 | down |
| ENSG00000238133 | MAP3K20-AS1 | -5.574161211 | down |
| ENSG00000272807 | ENSG00000272807 | -5.574161211 | down |
| ENSG00000235529 | AGAP1-IT1 | -5.574161211 | down |
| ENSG00000124253 | PCK1 | -5.574161211 | down |
| ENSG00000186998 | EMID1 | -5.574161211 | down |
| ENSG00000286492 | ENSG00000286492 | -5.574161211 | down |
| ENSG00000273455 | ENSG00000273455 | -5.574161211 | down |
| ENSG00000250412 | KLHL2P1 | -5.574161211 | down |
| ENSG00000278900 | ENSG00000278900 | -5.574161211 | down |
| ENSG00000289267 | ENSG00000289267 | -5.574161211 | down |
| ENSG00000232385 | RPS3AP25 | -5.574161211 | down |
| ENSG00000253944 | MTUS1-DT | -5.574161211 | down |
| ENSG00000181790 | ADGRB1 | -5.574161211 | down |
| ENSG00000232104 | RFX3-DT | -5.574161211 | down |
| ENSG00000279422 | ENSG00000279422 | -5.574161211 | down |
| ENSG00000102001 | CACNA1F | -5.574161211 | down |
| ENSG00000285018 | ENSG00000285018 | -5.574161211 | down |
| ENSG00000273002 | ARHGEF2-AS2 | -2.307180697 | down |
| ENSG00000183914 | DNAH2 | -2.307180697 | down |
| ENSG00000284690 | CD300H | -2.307180697 | down |
| ENSG00000233654 | NEMP2-DT | -2.307180697 | down |
| ENSG00000136867 | SLC31A2 | -2.307180697 | down |
| ENSG00000224975 | INE1 | -2.307180697 | down |
| ENSG00000260293 | ENSG00000260293 | -1.390601727 | down |
| ENSG00000288860 | ENSG00000288860 | -1.390601727 | down |
| ENSG00000143320 | CRABP2 | -1.105267587 | down |
| ENSG00000245556 | SCAMP1-AS1 | -1.011212192 | down |
| ENSG00000260329 | TMEM263-DT | -1.434276255 | down |
| ENSG00000269439 | PGLS-DT | -1.434276255 | down |
| ENSG00000286219 | NOTCH2NLC | -1.486918417 | down |
| ENSG00000286918 | ENSG00000286918 | -1.988435705 | down |
| ENSG00000172458 | IL17D | -1.988435705 | down |
| ENSG00000058335 | RASGRF1 | -1.988435705 | down |
| ENSG00000275888 | ENSG00000275888 | -1.988435705 | down |
| ENSG00000273355 | ENSG00000273355 | -1.988435705 | down |
| ENSG00000101197 | BIRC7 | -1.988435705 | down |
| ENSG00000240057 | NEPRO-AS1 | -1.988435705 | down |
| ENSG00000132394 | EEFSEC | -1.988435705 | down |
| ENSG00000254343 | ENSG00000254343 | -1.988435705 | down |
| ENSG00000255145 | STX17-DT | -1.988435705 | down |
| ENSG00000261188 | TFIP11-DT | -1.067343582 | down |
| ENSG00000254389 | RHPN1-AS1 | -1.067343582 | down |
| ENSG00000256008 | CABP1-DT | -1.551655143 | down |
| ENSG00000246898 | LINC00920 | -1.551655143 | down |
| ENSG00000278709 | NKILA | -1.551655143 | down |
| ENSG00000114541 | FRMD4B | -1.551655143 | down |
| ENSG00000280187 | ENSG00000280187 | -1.174132966 | down |
| ENSG00000127863 | TNFRSF19 | -1.633290605 | down |
| ENSG00000242082 | SLC5A4-AS1 | -1.559775209 | down |
| ENSG00000185664 | PMEL | -1.792054866 | down |
| ENSG00000119725 | ZNF410 | -1.792054866 | down |
| ENSG00000124074 | ENKD1 | -1.792054866 | down |
| ENSG00000289021 | ENSG00000289021 | -1.792054866 | down |
| ENSG00000266783 | ENSG00000266783 | -1.792054866 | down |
| ENSG00000266743 | ENSG00000266743 | -1.792054866 | down |
| ENSG00000267372 | ENSG00000267372 | -1.792054866 | down |
| ENSG00000124097 | HMGB1P1 | -1.792054866 | down |
| ENSG00000226471 | ENSG00000226471 | -1.792054866 | down |
| ENSG00000198624 | CCDC69 | -1.792054866 | down |
| ENSG00000280916 | FOXCUT | -1.792054866 | down |
| ENSG00000232860 | SMG7-AS1 | -1.657776724 | down |
| ENSG00000270344 | POC1B-AS1 | -1.657776724 | down |
| ENSG00000249345 | LINC02405 | -1.657776724 | down |
| ENSG00000269947 | ENSG00000269947 | -1.657776724 | down |
| ENSG00000231274 | SBK3 | -1.657776724 | down |
| ENSG00000248866 | USP46-DT | -1.230733794 | down |
| ENSG00000183309 | ZNF623 | -1.108455897 | down |
| ENSG00000181513 | ACBD4 | -1.038775457 | down |
| ENSG00000231312 | MAP4K3-DT | -1.038775457 | down |
| ENSG00000188177 | ZC3H6 | -1.01037961 | down |
| ENSG00000225778 | PROSER2-AS1 | -1.276656068 | down |
